# Broadband single molecule SERS detection designed by warped optical spaces

**DOI:** 10.1038/s41467-018-07869-5

**Published:** 2018-12-21

**Authors:** Peng Mao, Changxu Liu, Gael Favraud, Qiang Chen, Min Han, Andrea Fratalocchi, Shuang Zhang

**Affiliations:** 1 School of Physics and Astronomy, University of Birmingham, Birmingham, B15 2TT United Kingdom; 2 College of Electronic and Optical Engineering & College of Microelectronics, Nanjing University of Posts and Telecommunications, Nanjing, 210023 China; 3 PRIMALIGHT, Faculty of Electrical Engineering, Applied Mathematics and Computational Science, KAUST, Thuwal, 23955-6900 Saudi Arabia; 4 National Laboratory of Solid State Microstructures, College of Engineering and Applied Sciences and Collaborative Innovation Centre of Advanced Microstructures, Nanjing University, Nanjing, 210093 China

## Abstract

Engineering hotspots is of crucial importance in many applications including energy harvesting, nano-lasers, subwavelength imaging, and biomedical sensing. Surface-enhanced Raman scattering spectroscopy is a key technique to identify analytes that would otherwise be difficult to diagnose. In standard systems, hotspots are realised with nanostructures made by acute tips or narrow gaps. Owing to the low probability for molecules to reach such tiny active regions, high sensitivity is always accompanied by a large preparation time for analyte accumulation which hinders the time response. Inspired by transformation optics, we introduce an approach based on warped spaces to manipulate hotspots, resulting in broadband enhancements in both the magnitude and volume. Experiments for single molecule detection with a fast soaking time are realised in conjunction with broadband response and uniformity. Such engineering could provide a new design platform for a rich manifold of devices, which can benefit from broadband and huge field enhancements.

## Abstract (web summary)

In standard SERS the probability for the molecules to reach tiny hotpot regions is low. Here, the authors introduce an approach based on warped spaces that offers a strategy to manipulate hotspots of metallic nanostructures, resulting in large broadband enhancements in both the magnitude and the volume size.

## Introduction

Achieving biochemical sensing with both ultrafast speed and detection limits down to single-molecule level is highly desirable in a plethora of applications, and in particular for those related to real-time environmental monitoring and pathogens and protein recognition for disease diagnosis. Surface-enhanced Raman scattering (SERS) spectroscopy is one of the most powerful analytical strategies used to date for the identification of chemical fingerprints under ambient conditions, by virtue of greatly enhanced inelastic Raman scattering from minute amounts of substance deposited near nanostructured metallic surfaces^1–4^. Many different SERS systems have been developed to achieve a large enhancement factor (EF) and good sensitivity, ranging from nanoparticle (NP) aggregate/oligomer^5–21^ to 2D/3D structured surfaces^22–27^ and hybrid structures^28–34^. Despite obtaining a high EF level beyond 10^7^, a comparatively long soaking time for sample preparation is needed, ranging from one hour to tens of hours^18,22,23,25,28,30^, owing to the tiny volume of regions with strong electromagnetic energy localisation. The effective volume only accounts for a tiny fraction of the total volume of the real device^6,12,17^, limiting the probability for the analytes to reach the region where energy hotspots exist. Such a slow process substantially hinders SERS in all practical applications that require stringent requirement on both sensitivity and time response, ranging from environment monitoring to hazardous pollutants and disease diagnosis in clinics. In this article, inspired by the recent development in transformation optics (TO), we design and implement a SERS spectroscopy system that possesses a large broadband sensitivity and fast time response at the same time. By virtue of the equivalence of light propagation between media containing gradients in optical properties and warped geometries of spacetime^35–39^, we here propose to leverage the curvature of the space to engineer electromagnetic hotspots that can substantially enhance both the effective volume and the magnitude of the field, hence improving the Raman response of an analyte in a broad spectrum. As a result of this approach, the number of molecules that can access the region with high EF significantly increases, thus enabling fast SERS detection with high sensitivity. In our samples realised with gas-phase cluster beam deposition^40^, we observed a 20-fold enhancement of Raman signal compared to a reference flat substrate, reporting single-molecule detectability with short soaking time of 60 s. The measured EF reaches average value beyond 10^8^ inside a broadband in visible, and exhibits comparable repeatability and uniformity^34,41,42^. Employing warped spatial geometries as an additional degree of freedom in hotspot engineering may offer new strategies towards design principles for applications also in nonlinear optics^43^, plasmonic lasers^44^ and hot-electrons^45^.

## Results

### Sample design and nanofabrication

**Fig. 1:**
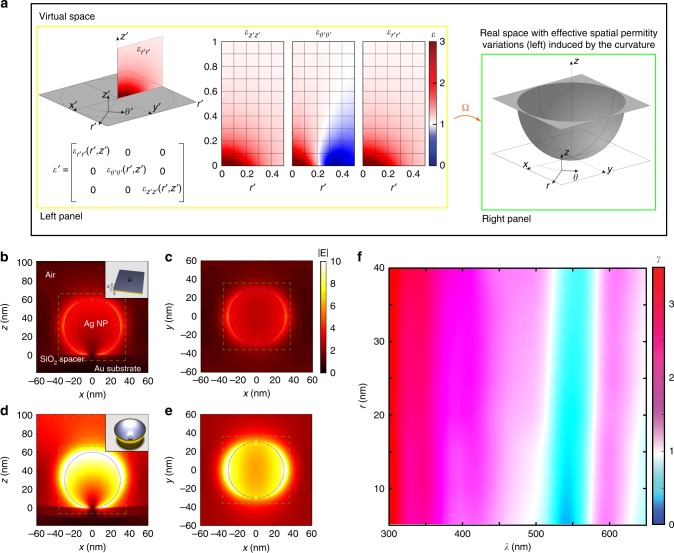
Curvature-induced broadband field enhancement. **a** Left panel: A desired but unrealistic distribution of refractive index of air above a flat substrate that can achieve light confinement. The permittivity *ε*′ is presented in cylindrical coordinates (*r*′, *θ*′, *z*′). Right panel: Transformed structure obtained by applying a coordinate mapping (*x*, *y*, *z*) = Ω(*x*′, *y*′, *z*′), with permittivity above the substrate equal to 1. The spatial variation of *ε*′ in left panel is mapped to the spatial curvature without changing the dynamics of the electromagnetic waves. **b**, **c** 3D FDTD simulations for a Ag nanoparticle on flat Au substrate with 10 nm thick silica spacer in between. The inset in **b** is a schematic of the setup. **d**, **e** 3D FDTD simulations for a Ag nanoparticle on warped Au substrate. The inset in **d** is a schematic of the setup. A prominent field enhancement is demonstrated due to the effective gradient of permittivity described in **a**. **f** A summary of the averaged field enhancement factor $\gamma = \overline {|{\mathbf{E}}|} _{{\mathrm{curv}}}{\mathrm{/}}\overline {|{\mathbf{E}}|} _{{\mathrm{flat}}}$ for different wavelength *λ* and particle radius *r*. A broadband enhancement is illustrated for all nanoparticles with different sizes, owing to the wavelength independence permittivity gradient induced by curvature

The starting point of our design is from a very simple configuration, represented by a plasmonic NP on top of a flat substrate in contact with air. In this configuration the electromagnetic field cannot be confined horizontally, owing to the homogenous refractive index *n*_air_ = 1. Hypothetically, if an additional spatial variation of refractive index is induced in the vicinity of the substrate, the electromagnetic energy could be trapped and consequently boost the electromagnetic energy in the proximity of the NP (see more details in Supplementary Note 1). However, inducing a prominent variation of *n*_air_ (such as the one shown in left panel of Fig. 1a) seem impossible to realise for a homogeneous material. However, with the aid of TO, we can create an equivalent structure that can give the illusion to light to propagate in such a material with a spatially varying refractive index, thus overcoming this challenge and inducing an extremely high energy localisation. This task is accomplished by warping the space (*x*′, *y*′, *z*′) by a coordinate transformation (*x, y, z*) = Ω (*x*′, *y*′, *z*′), as illustrated in the right panel of Fig. 1a. In the real (transformed) space (*x, y, z*), the dynamics of light is described by the same set of Maxwell’s equations that models photon dynamics in the original (virtual) space (*x*′, *y*′, *z*′), but with a new homogeneous medium above the substrate with refractive index *n*(*x, y, z*) = 1. The left and right configurations of Fig. 1a are optically equivalent. Details on the demonstration of this conditions can be found in refs. ^38,39^, as well as in the Supplementary Note 2.

The warped substrate with a constant curvature *κ* (Fig. 1c) is a practical structure, which can strongly localise the electromagnetic waves due to the induced effective refractive index gradient. For the quantitative demonstration of this effect, we implement 3D full-wave simulations based on the finite difference method in time domain (FDTD). Figure 1b–e compare the magnitude of the electric field |**E**| for a metallic NP lying on the top of (a, b) flat and (c, d) warped substrate under normal illumination of a monochromatic light with wavelength *λ* = 345 nm. We select the radius of the Ag NP as *r* = 30 nm, and the curvature of the substrate as *κ* = 2 µm^−1^. A silica spacer with 10 nm thickness is inserted between the NP and the Au substrate. A hotspot is formed around the NP with enhanced electric field in a quite limited area (Fig. 1b, c). Figure 1d, e illustrates the situation when a NP lies on a warped surface formed by an arc with inscribed angle of *π*. Interestingly, the electric field around the NP experiences an immense enhancement in the warped substrate to form a larger and brighter hotspot, matching our qualitative prediction from TO in Fig. 1a. Quantitative comparison of the size of hotspot between flat and warped substrate can be found in Supplementary Note 4. A more quantitative comparison is obtained by calculating the spatially averaged electric field $\overline {|{\mathbf{E}}|} = \mathop {\int}\limits_A {\left| {\mathbf{E}} \right|{{\mathrm{d}}x{\mathrm{d}}y{\mathrm{d}}z/}\mathop {\int}\limits_A {{{\mathrm{d}}x{\mathrm{d}}y{\mathrm{d}}z}} }$, where *A* is the volume with refractive index *n*(*x, y, z*) = *n*_air_ covered by a square region of length 2.4*r* (green dashed lines in Fig. 1a, c). The ratio between the averaged field in warped and flat substrate$\gamma = \overline {|{\mathbf{E}}|} _{{\mathrm{curv}}}{\mathrm{/}}\overline {|{\mathbf{E}}|} _{{\mathrm{flat}}}$ provides a measure of the averaged field EF. We achieved an averaged EF of around 300% for the electric field in the air around the NP in warped space. We further implemented a number of simulations with different NP radius *r* and wavelength *λ* for a comprehensive investigation and the results are shown in Fig. 1f. Our warped structure achieves broadband enhancement in the visible region for all NPs with different radius *r* despite the fluctuations of the averaged electric fields for NPs with different plasmonic resonances (see Supplementary Note 3 for more details). This remarkable enhancement effect results from the fact that the gradient of *n*_eff_ induced by curvature is independent of the wavelength^37^. The situation when the NP deviates from the centre of nanobowl (NB) is investigated in the Supplementary Note 5.

**Fig. 2:**
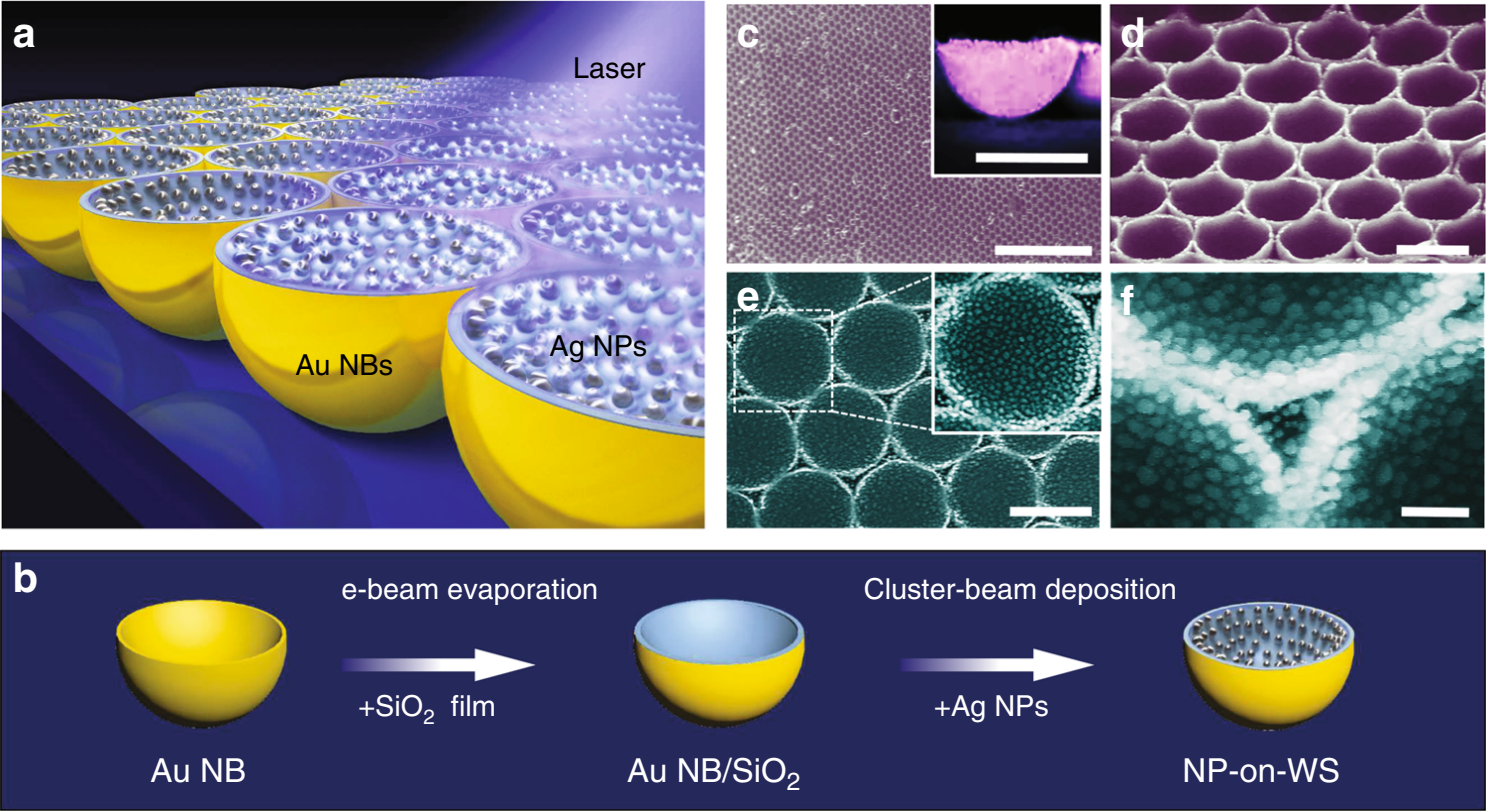
TO-inspired SERS design and fabrication. **a** Schematic illustration of SERS substrate configuration for nanoparticles on warped substrate (NP-on-WS) hierarchical nanostructure, with nanoparticle acting as nanolens to focus the light to its vicinity and NBs providing additional light confinement induced by the curvature. **b** The fabrication process of hierarchical NP-on-WS nanostructures. A silica film is deposited on Au NB fabricate from silica template. Then a monolayer of nanoparticles is uniformly deposited inside NB by gas-phase deposition. **c** Low-magnified SEM images of the hexagonal close-packed Au NB array. The scale bar represents 15 µm. The inset shows a horizontal view of Au NBs with scale bar representing 1 µm. **d** Tilted-view SEM images of the hexagonal close-packed Au NB array. The scale bar represents 1 µm. **e** Low magnification of experimental realisation of NP-on-WS nanostructure in **a**. The scale bar represents 1 µm. **f** High magnification of experimental realisation of NP-on-WS nanostructure in **a**. The scale bar represents 200 nm

Based on this mechanism of warped spatial coordinates, we designed a SERS spectroscopy device by applying our model to a warped 3D structure with a constant radial curvature in the space. Multiple NPs are utilised to improve the localisation of light by interparticle coupling, with hotspots further enhanced by the warped substrate (see Supplementary Note 6 for more details). Figure 2a is a schematic illustration of the designed SERS system-a 3D hierarchical nanostructure of NPs in warped substrate (NP-on-WS) formed by NBs. Our hybrid SERS substrate is composed of a monolayer of Ag clusters deposited inside highly-ordered Au NB arrays. Inside NBs, each NP strongly confines light around, significantly enhancing the sensitivity of SERS in the vicinity of the NP.

The substrate is fabricated by combining template metal deposition and gas-phase cluster beam deposition, as shown schematically in Fig. 2b (see Methods for more details). Figure 2c,d shows scanning electron microscope (SEM) images of Au NBs fabricated from silica template, revealing close-packed arrays that can efficiently provide NP with warped spaces. To obtain a more precise control of the number and location of the NPs, we perform gas-phase cluster beam technique instead of colloidal self-assembly, to obtain a homogeneous coverage with the maximisation of the density of the hotspots in the sensor. Figure 2e, f shows typical SEM images of the final NP-on-WS hierarchical structures, with Ag NPs uniformly distributed on the entire inner surface of the Au NBs. We implemented a statistical analysis of the diameter distribution of Ag NPs based on SEM images, acquiring a logarithmic normal distribution with a mean value of 48 nm and a standard deviation of 10.4 nm (more details in Supplementary Note 8). Despite the fluctuation of radii, all NPs reach similar level of field enhancement by the curvature as shown in Fig. 1e.

### Warped space SERS device characterisation

**Fig. 3:**
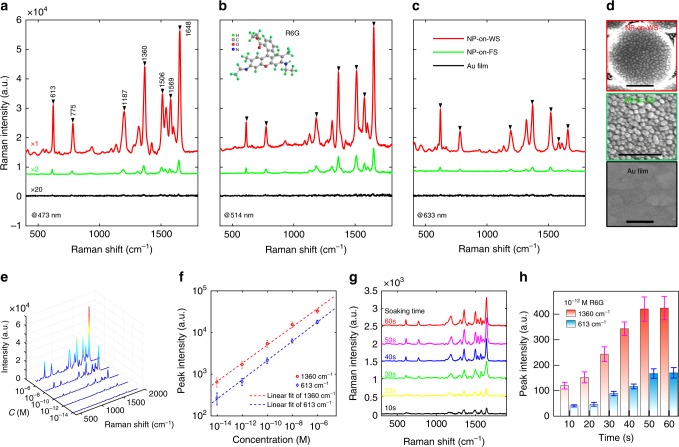
TO-inspired SERS spectroscopy with broadband response, ultrahigh sensitivity and fast response on NP-on-WS structure. **a**–**c** The SERS spectra are shown for the NP-on-WS sample with different pump laser at (**a**) 473 nm, (**b**) 514 nm and (**c**) 633 nm. The signals from the reference (flat Au film) and the control (NP-on-FS) are also demonstrated. **d** The corresponding SEM images of the NP-on-WS sample (top), NP-on-FS control (middle) and the reference (bottom). The scale bar represents 500 nm. **e** SERS spectra of R6G with five different concentrations. Distinguishable peaks are demonstrated even at ultra dilute concentration of 10^−12^ M. The measurement for each R6G concentration is performed at five different locations of the NP-in-NB substrate. **f** The SERS intensity of R6G peaks at 613 cm^−1^ (red) and 1360 cm^−1^ (blue) as a function of the molecular concentration in log–log scale. Error bars indicating the standard deviation of the peak intensities over different positions. A prominent linear relationship is presented with the fit (dashed lines), demonstrating the potential for label-free quantitative sensing. **g** SERS spectra of R6G over substrates with different soaking times, with concentration of 10^−12^ M. Raman signals at 1360 cm^−1^ are observed unambiguously even within 10 s. **h** The SERS intensity of R6G peaks at 613 and 1360 cm^−1^ as a function of the soaking time. Error bars represent the mean value of three replicate samples

We investigate the sensing capability of the prepared NP-on-WS hierarchical nanostructure array in detecting a typical organic analyte—R6G. We immersed the samples into methanol solution of R6G for a short period (60 s). Details of the sample preparation and SERS measurements are shown in the methods. We implement the SERS measurements at different wavelengths to investigate the broadband field enhancement predicted in the previous section. Figure 3a–c illustrates the Raman spectra with pump lasers at 473, 514 and 633 nm respectively, for NP-on-WS, NPs on flat substrate (NP-on-FS) as a control, and a reference of Au film with SEM images presented in Fig. 3d. The positions of the characteristic peaks of R6G including 613, 775, 1187, 1309, 1360, 1506, 1569 and 1648 cm^−1^ are in agreement with previously reported studies^6,19,23,30,34^. For quantitative analysis, we compute the globally averaged field EF based on the data from Fig. 3a–c. Rather than making the standard assumption that Raman scattering originates only from a monolayer of molecules that cover the effective surface of the nanostructures^15,22,25,29–32,34^, we here use a measure characterised by the averaged EF that originates for all the molecules in the system. This measure provide a better matching of a realistic situation and also eliminates any inaccuracy resulting from the estimation of the active surface area of each nanostructure and the cross-sections of the anisotropic molecules. The globally averaged field EF is defined as a simple normalisation of Raman intensity^46^:

$$\overline {EF} = \frac{I}{{I_0}}\frac{{C_0}}{C}\frac{{P_0}}{P}{,}$$

where *I* is the Raman intensity, *C* the molar concentration of the solution and *P* the laser power respectively. *I*_0_, *C*_0_ and *P*_0_ are the corresponding values of the baseline with a pure Au film. Remarkably, we experimentally achieve a broadband *EF* beyond 10^8^ at 1360 cm^−1^ for NP-on-WS, as summarised in Table 1. To disentangle the impact of absorption/scattering cross-section of the NPs and analytes at different wavelengths, we define a parameter *α*_I_ = *I*_NP-on-WS_*/I*_NP-on-FS_ that purely represents the enhancement due to the curved substrate, as demonstrated in Table 1. *I*_NP-on-WS_ and *I*_NP-on-FS_ are the Raman peak intensities at 1360 cm^−1^ for NP-on-WS and NP-on-FS under same molecule concentration and laser power. The spectra for 785 nm laser can be found in Supplementary Note 9. A prominent improvement is observed from the value of *α*_I_, implying the corporative effect of both enlarged size and enhanced intensity of the hot region around the NP induced by the warped substrate. The curved substrate not only increases the NPs density by a factor of 1.6 (see Supplementary Note 15), more importantly, induces the effective gradient of refractive index to achieve 20× boost at 473, 633 and 785 nm. The degradation of the *α*_I_ at 514 nm pump qualitatively agrees with the simulation results in Fig. 1f for |**E**| of single NP at bottom. However, the enhancement still exists due to the contributions from the NPs away from the centre of the bowl. Detailed explanation with simulation on the fourth power of surface electric field that proportional to the Raman signal intensity can be found in Supplementary Note 5. Such broadband response provides flexibility for selecting the pump laser to maximise the cross-section of the analyte and consequently further improving the sensitivity. To demonstrate the generality of the curved induced field enhancement, we also investigate the SERS system with different NP densities, showing a nearly homogeneously enhancement with the value of *α*_I_ around 20. The EF estimation with single-layer coverage is also investigated with similar value of *α*_I_. See Supplementary Note 14 for more details. Additional evidence for the curvature-induced field enhancement is demonstrated in Supplementary Note 17.

**Table 1:** A summary of *EF* at 1360 cm^−1^ and *α*_I_ for four different wavelengths

	473 nm	514 nm	633 nm	785 nm
*EF* @1360 cm^−1^ (NP-on-WS)	1.11 × 10^9^	1.20 × 10^9^	0.54 × 10^9^	1.16 × 10^9^
*EF* @1360 cm^−1^ (NP-on-WS)	0.54 × 10^8^	2.47 × 10^8^	0.24 × 10^8^	0.50 × 10^8^
*α*_*I*_ (*I*_NP-on-WS_/*I*_NP-on-FS_)	20.4	4.8	22.2	23.2

To demonstrate the potential application of our warped space sensor as an ultra-sensitive SERS chemical detector, measurements are performed for samples immersed in R6G solutions of different concentrations from 10^−6^ M down to 10^−14^ M, for only 60 s. Figure 3e presents typical spectra for different R6G concentrations. In spite of the intensity decrease at more dilute concentrations, unambiguous signatures can be straightforwardly distinguished in the SERS spectra even at the extremely low concentration of 10^−12^ M. The detection limit can be further improved by other means, such as increasing the integration time and/or the laser power. For the quantitative analysis, we select two Raman resonances at 613 and 1360 cm^−1^, which correspond to the in-plane vibration of C–C–C bonds and deformed C–C bonds, respectively^23^. The dependence of peak intensities on R6G concentration is plotted in Fig. 3f. In a broad concentration range, the figure demonstrates a good linear relationship in log–log scale between the concentration and Raman intensity with close-to-unit coefficient of determination *R*^2^ (0.9801 for 613 and 0.9838 for 1360 cm^−1^), providing the potential for label-free quantitative detection of chemicals.

We investigated the temporal dependence of Raman scattering utilising extremely diluted solution with concentration of 10^−12^ M, as shown in Fig. 3g–h. In Fig. 3g, SERS spectra of R6G are plotted with different soaking times. Strong Raman signals are observed unambiguously even within 10 s, as verified by the dependence of the peak intensities over the time in Fig. 3h. Remarkably, our SERS system does not require long preparation time, benefited from the increased probability of molecules reaching the enlarged active region.

**Fig. 4:**
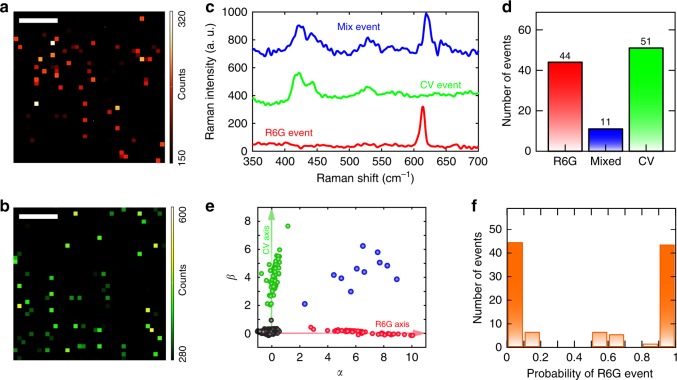
Bi-analyte single-molecule detection base on MPCA. **a** 2D Raman mapping of the peak intensity at a 613 cm^−1^ corresponding to R6G. The scale bar represents 5 µm. **b** 2D Raman mapping of the peak intensity at a 414 cm^−1^ corresponding to CV. The scale bar represents 5 µm. **c** Typical spectra for bi-analyte analysis. The details of all the spectra can be found in the Supplementary Note 10. **d** Histogram of occurrences of pure R6G, pure CV and mixed events. **e** Coefficient (*α*,*β*) plot of the spectra based on the eigenvectors from MPCA. The axes represent two principle eigenvectors that corresponds to the R6G (red) and CV (green) events. The points in between and near the origin corresponds to the mixed events (blue) and null events (black) respectively. **f** Single-molecule probability histogram derived from MPCA, providing a clear sign for sensitivity at single-molecule level

To experimentally confirm the single-molecule sensitivity for our system, we apply bi-analyte approach that is based on spectroscopic contrast between two different kinds of SERS-active molecules^47–50^. We select the two molecules as R6G and crystal violet (CV), which are mixed and deposit on the SERS substrate with soaking time of 60 s. We obtain 1600 spectra from 2D Raman scanning on substrate within a square area of 20 µm length, as illustrated in Fig. 4a, b. The intensity in Fig. 4a is the mapping at 613 cm^−1^ corresponding to peaks of R6G while the intensity in Fig. 4b is the mapping at 414 cm^−1^ for peaks of CV. Figure 4c demonstrates the typical bi-analyte spectra for three different events. In contrast to more concentrated solutions of the two analytes which should always yield different mixed spectra, the collected spectra are dominated by either one analyte (red/green solid line) or mixture of one R6G and one CV molecule (blue solid line), or no molecules detected at all (null event). The ratio among R6G: mixed: CV is 4: 1: 4.8 (as shown in Fig. 4d), matching the ratio previously reported for single-molecule sensitivity^47,48^. Besides the analysis above based on empirical event counting, we also implement a more rigorous statistical analysis based modified principal component analysis (MPCA)^49–51^, whose results are independent of the ratio between the number of molecules and the nanostructures and cross-section difference of the active molecules. MPCA typically uses two principal components to represent the data set (the spectra), as shown in Fig. 4e. Two batches of spectra are clearly classified as R6G (red circles) and CV (green circles) events correspondingly, with mixed events (blue circles) between the axes and null event locating near the origin (black circles). The probability of single-molecule signature is plotted in the form of a histogram where the noise (null events) is excluded, as shown in Fig. 4f. Dominant and equal contributions from the single-dye-signal events at *p* ∼0 (CV events) and *p* ∼1 (R6G events) is observed, verifying the single-molecule detection regime for our system. More details for the MPCA approach can be found in the Supplementary Note 10.

Besides sensitivity and rapid time responses, the uniformity of the active substrate is another beneficial factors for a desired SERS spectroscopy in practical applications. We performed Raman mapping our SERS systems and acquire reproducible and stable spectra with a relative standard deviation of 6.82% at the concentration of 10^−6^ M and 14.08% at the concentration of 10^−12^ M, with more details in the Supplementary Note 11.

## Discussion

By exploiting the equivalence of light propagation between spatio-temporal geometries and materials with variation of refractive index, we designed and characterised a SERS device based on warped spaces that strongly enhance broadband electromagnetic energy over a relatively large spectral region. Such warped induced broadband field enhancement is experimentally observe in SERS spectroscopy experiments with NPs of different sizes deposited on warped substrate fabricated hierarchically with gas-phase deposition, which shows a 20-fold enhancement compared with a classic control system made by NPs deposited on a flat substrate. In general, a trade-off between the time response and sensitivity always exists for the SERS detection, owing to the fact that it always takes some time for the analyte to achieve the hotspots. The substrate based SERS with long soaking time can achieve high EF for single-molecule level detection while the NP based system can be readily merged with the analyte even for in situ detection^11,52,53^ at the cost of reduced sensitivity. Taking advantage of the power from TO, we mitigate the stringent constraint between time and detectability, achieving single-molecule detection with only 60 s soaking time. Besides, owing to the versatility of both fabricating different types of NPs with gas-phase deposition technology and the pump laser wavelength, our system can be feasibly adapted for label-free detection of many different types of molecules and analytes. This can be particularly applied to proteins such as EGFR and HER2/neu, with the size beyond tens of nanometres, which are used in early cancer screening diagnoses. See more details of SERS protein detection in Supplementary Note 16.

Not limited to biochemical sensing, hotspots engineering with warped spaces with high-broadband sensitivity and large volume can boost the development of many different applications of crucial importance in many fields, including nonlinear harmonic generation, plasmonic laser and hot-electrons, where new structures can be engineered by equivalent systems where the existence of specific media is substituted by suitably defined warped spatial geometries. As discussed in this specific example, this approach can overcome challenges that would otherwise seem impossible to address, thus realising a class of high performing devices for a manifold of real-world applications. Meanwhile, for more sophisticated structures with multilayer structures, the integrating the device into the curved substrate may be a technical challenge to overcome. And the enhancement variation across the spectrum may require additional judicious design to achieve the optimal broadband performance.

## Methods

### Numerical calculations of curvature-induced field enhancement

FDTD calculations with commercial software (FDTD solution, Lumerical Inc.) are used to simulate the linear response of silver NPs on flat/warped Au substrate with 10 nm thick silica spacer. The optical constants of gold and silver are taken from ref. ^54^. For the warped case, curvature of the substrate is chosen as constant value *κ* = 2 µm^−1^, corresponding to a circle with radius of 500 nm. And the inscribed angle of the substrate is select as *π*, i.e., half of the circle. An electric field is applied with polarisation along *x*.

### Sample fabrication

Au NB array was prepared by the template metal deposition method. Gold layer with thickness of 50 nm was deposited by vacuum evaporation on a sacrificial 2D hexagonally close-packing colloidal crystal template, making the microbeads hemispherically covered by gold. Then the silica template was etched by using hydrogen fluoride acid to leave the Au NBs in the solution. And the upward interconnected Au NB arrays can be prepared via a transferring process. A 10 nm thick SiO_2_ thin film uniformly covered the entire surfaces of the Au NBs by performing e-beam evaporation. Then, gas-phase cluster beam deposition process is used to deposit Ag NPs on the inner wall surface of Au/SiO_2_ NB structure. A silver plate with high purity (99.99%) is used as the sputtering target. A DC power supply was used for the sputtering of Ag target in argon gas ambient with a pressure of ∼100 Pa, maintained by passing argon gas to the liquid nitrogen-cooled aggregation tube. Sputtered Ag atoms lost energy by colliding with the cooled argon gas in the aggregation tube and formed NPs. The NPs were swept by the gas stream into high vacuum through a nozzle and a skimmer, forming a collimated NP beam with a high speed of ∼1000 m/s, and then deposited on the surface of substrates. The deposition was carried out at a rate of 0.5Å /s for 10 min. In situ annealing was carried out for 10 min at 150 °C. More details of the fabrication process can be found in Supplementary Notes 7 and 8.

### SERS measurement

For Raman measurements, SERS substrates were soaked in R6G-ethanol, CV-ethanol and R6G/CV-ethanol solution for different time and then remove them from the solution vertically and then dried under flowing *N*_2_. For quantitative estimation of the EF of the NP-on-WS nanostructure, a 100 mM R6G-in-methanol solution was used to prepare a reference sample. For the reference sample, a drop (around 10 µL) of 100 mM R6G-in-methanol solution was dispersed onto a flat Au film surface supported with silica slice. For Raman spectrum measurement lasers with different wavelengths (473, 514, 633 and 785 nm) (through a 100× objective lens) was employed with a beam spot size of 700 nm in diameter. The Raman spectra are recorded from an upright-configured confocal microscopy (NT-MDA NTEGRA SPECTRA and HR Evolution HORIBA JOBIN YVON). The laser power used is 1, 0.1, and 0.01 mW for different structures. More details of Raman characterisation can be found in Supplementary Note 9. Raman mappings are performed in sample-scanning mode with a step of 0.5 µm per point and a dwell time of 1 s per point.

## Supplementary information

Supplementary Information

## Data Availability

The data that support the findings of this study are available from the corresponding author upon request.
