# Supplementary material for: Broadband single molecule SERS detection designed by warped optical spaces
Source: Nat Commun. 2018 Dec 21;9:5428. doi: 10.1038/s41467-018-07869-5 (PMC6303368; doi:10.1038/s41467-018-07869-5)
Supplement: Supplementary file 1 — Supplementary Information [file 41467_2018_7869_MOESM1_ESM.pdf]

Supplementary Information for ”Broadband single molecule SERS detection designed by warped optical spaces”

Mao et. al.

November 17, 2018

Supplementary Note 1: field enhancement in a virtual space with permittivity gradient

Our original idea is to enhance the hotspot through the additional confinement induced by the permittivity gradient in virtual space. Here we demonstrate such effect, as summarised in Supplementary Figure 1. To show the concept, we use 2D simulations for the sake of simplicity.

We choose three different types of spatial gradient of the permittivity ϵ as shown in Supplementary Figure 1 for the generality. The ϵ varies along the radius of the circle centred at origin. And a homologous media with $\epsilon = 1$ is also included as a reference. Supplementary Figure 1b illustrate the electric field in x direction without any nanoparticle. The electric field experiences an enhancement owing to the existence of the spatial gradient of ϵ. In Supplementary Figure 1c, we position a silver nanoparticle and illustrate the corresponding magnitude of the electric field. We observe a general enhancement in both the size and the intensity of the hotspots generated around the nanoparticles, even with different gradients.

The variation of refractive index in the virtual space can be mapped to real space of curved surface with homogenous refractive index by transformation optics (more details in the following section). For the sake of the fabrication, we choose a more complex distribution of ϵ (as shown in the Fig. 1a in the main text), which can simply be mapped to a surface with constant curvature. A comparison for such ϵ distribution is also demonstrated in Supplementary Figure 2, demonstrating a definite field enhancement.

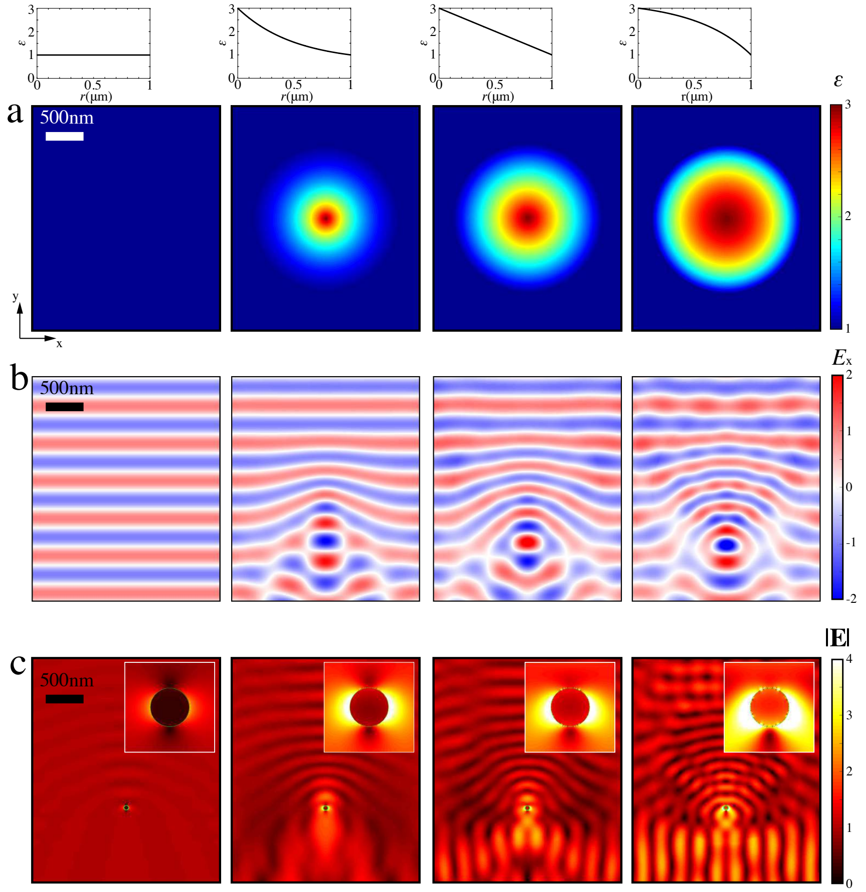

Supplementary Figure 1. **a** The spatial gradient of ϵ. Three different types of gradient is introduced from column 2 to 4, while column 1 is a reference with homogenous ϵ. The top insets show the ϵ variation along the radius r of a circle centred at origin. **b** The corresponding spatial distribution of E_x. A plane wave with $\lambda = 500nm$ is launched along -y. **c** The corresponding spatial distribution of $|E|$ with a Ag nanoparticle. The top insets are zoomed-in pictures to illustrate the hotspots formed around the nanoparticles.

Supplementary Note 2: Transformation optics

This section details how is established the equivalence between the material with inhomogeneous refractive index distribution and the bowl shaped geometry, both represented on Fig. 1a of the main text, and how the refractive index distribution associated to this particular shape is computed. This result comes from Transformation Optics (TO) [18, 14], which was initially presented to design invisibility cloaks and relies on the form invariance of the Maxwell equation under coordinate transformations. This theoretical frame allows one to establishes the equivalence between two configurations linked by a coordinate transformation. It is broadly used to realize a desired ideal configuration that is hard (or impossible) to fabricate, by physically implementing a more practical equivalent configuration obtained through coordinate transformation.

In the case of the left panel of Fig. 1a, we consider an ideal configuration in a virtual space equipped with the curvilinear coordinates (x', y', z'). In this space the effective medium is characterized by a complex inhomogeneous distribution of permittivity ε' and permeability μ', which induce an inhomogeneous refractive index depending on the considered polarization and propagation direction. These parameters are in this case diagonal tensors when expressed in a polar basis. This configuration in the virtual space is completely equivalent to the configuration in the physical space with coordinates (x, y, z) depicted on the right panel of Fig. 1a, and on Fig. S1a. It is composed of an infinite flat surface having a half spherical cavity hole constituting a nanobowl.

Our approach reproduces the one presented in the chapter 1.5 of [11], that was proposed in order to flatten [19] a particular type of spherical lens called Lunerberg lens. It makes use of a 2D conformal mapping as a building block for a 3D quasiconformal transformation. Taking advantage of the cylindrical symmetry of the problem we consider a conformal mapping restricted to the cylindrical coordinates (r, z), while the θ coordinate is left unchanged:

$$r' = r'(r, z) \tag{1a}$$

$$\theta' = \theta \tag{1b}$$

$$z' = z'(r, z) \tag{1c}$$

This mapping transforms a cross section of the nanobowl (as on Fig. S1b) into a flat surface (Fig. S1c). The Jacobian of this conformal mapping writes

$$\mathsf{A}_{\mathrm{cm}} = \begin{bmatrix} \frac{\partial r'}{\partial r} & 0 & \frac{\partial r'}{\partial z} \\ 0 & 1 & 0 \\ \frac{\partial z'}{\partial r} & 0 & \frac{\partial z'}{\partial z} \end{bmatrix}, \tag{2}$$

which, owing to Transformation Optics formulas [18, 14], induces the following equivalent material properties in the virtual space:

$$\varepsilon' = \frac{\mathsf{A}_{\mathrm{cm}} \varepsilon \mathsf{A}_{\mathrm{cm}}{}^{\mathsf{T}}}{|\mathsf{A}_{\mathrm{cm}}|}, \qquad \mu' = \frac{\mathsf{A}_{\mathrm{cm}} \mu \mathsf{A}_{\mathrm{cm}}{}^{\mathsf{T}}}{|\mathsf{A}_{\mathrm{cm}}|}, \tag{3}$$

where ε and μ are the material parameters in the physical space (here we suppose $\varepsilon = \mu = 1$). Conformal mappings have the particularity that the two transformed coordinates are linked through the Cauchy-Rieman equations:

$$\frac{\partial r'}{\partial r} = \frac{\partial z'}{\partial z}, \tag{4a}$$

$$\frac{\partial r'}{\partial z} = -\frac{\partial z'}{\partial r}. \tag{4b}$$

Thanks to this particular relationship, the expression of the equivalent constitutive parameters in Eq. (3) reduces to a very simple form:

$$\varepsilon' = \mu' = \begin{bmatrix} 1 & 0 & 0 \\ 0 & \frac{1}{|\mathsf{A}_{\mathrm{cm}}|} & 0 \\ 0 & 0 & 1 \end{bmatrix}, \tag{5}$$

where the Jacobian reduces to

$$|\mathsf{A}_{\mathrm{cm}}| = \left(\frac{\partial r'}{\partial r}\right)^2 + \left(\frac{\partial r'}{\partial z}\right)^2 = \left(\frac{\partial z'}{\partial r}\right)^2 + \left(\frac{\partial z'}{\partial z}\right)^2. \tag{6}$$

The equivalent material is then characterized by the quantity $\frac{1}{|\mathsf{A}_{\mathrm{cm}}|}$, represented on Fig.S1c.

It has however to be noted that this expression holds in the vector basis induced by the coordinate system (a so-called coordinate basis) which is not orthonormal: the basis vector in the θ direction is given by:

$$\vec{e}_\theta = r\cos\theta\vec{e}_x + r\sin\theta\vec{e}_y, \tag{7}$$

where $(\vec{e}_x, \vec{e}_y, \vec{e}_z)$ is the cartesian basis in the (x, y, z) coordinate system. A similar result holds for the primed coordinate system and its induced based. As it is not natural to have these material parameters expressed in a non orthonormal basis, we rewrite the equivalent parameters formula in term of a transformation from a unit basis to another unit basis. This basically requires to apply a change of basis before applying the conformal mapping to go from a unit basis to the coordinate basis, and perform the inverse operation once the conformal mapping applied. The change of basis from the coordinate basis to the unit basis is performed through the change-of-basis matrix:

$$\mathsf{A}_{\mathrm{cu}} = \begin{bmatrix} 1 & 0 & 0 \\ 0 & r & 0 \\ 0 & 0 & 1 \end{bmatrix}, \tag{8}$$

while in the primed coordinates the opposite change of basis has to be performed through the matrix:

$$\mathsf{A}_{\mathrm{u'c'}} = \mathsf{A}^{-1}_{\mathrm{c'u'}} = \begin{bmatrix} 1 & 0 & 0 \\ 0 & \frac{1}{r'} & 0 \\ 0 & 0 & 1 \end{bmatrix}. \tag{9}$$

The operator of the whole transformation is therefore given by

$$\mathsf{A} = \mathsf{A}_{\mathrm{c'u'}}\mathsf{A}_{\mathrm{cm}}\mathsf{A}_{\mathrm{uc}} \tag{10a}$$

$$= \begin{bmatrix} \frac{\partial r'}{\partial r} & 0 & \frac{\partial r'}{\partial z} \\ 0 & \frac{r'}{r} & 0 \\ \frac{\partial z'}{\partial r} & 0 & \frac{\partial z'}{\partial z} \end{bmatrix} \tag{10b}$$

Applying the transformation rules (3) with this time the operator A leads to the final expression for the equivalent material constitutive parameters expressed in a classical cylindrical unit basis:

$$\varepsilon' = \mu' = \begin{bmatrix} \frac{r}{r'} & 0 & 0 \\ 0 & \frac{1}{|\mathsf{A}_{\mathrm{cm}}|}\frac{r'}{r} & 0 \\ 0 & 0 & \frac{r}{r'} \end{bmatrix}, \tag{11}$$

which are the one represented on Fig. 1 of the main text.

The determination of the conformal mapping $(r, z) \mapsto (r', z')$ itself is done following the same approach to one used on irregular or random surfaces [4, 10, 20]. This approach takes advantage of a very important property of conformal mappings that directly follows from the Cauchy-Riemann equations, which is that both coordinates satisfy a Laplace equation:

$$\frac{\partial^2 r'}{\partial r^2} + \frac{\partial^2 r'}{\partial z^2} = 0, \tag{12a}$$

$$\frac{\partial^2 z'}{\partial r^2} + \frac{\partial^2 z'}{\partial z^2} = 0. \tag{12b}$$

Despite the apparent simplicity of these Laplace equations, solving this direct problem is very difficult as an explicit boundary condition is not known: even though the shape of the target domain is known, the correspondence point by point at the boundary is still an unknown to be determined. It is however a lot simpler to solve the inverse problem, that is to compute the inverse transformation $(r', z') \mapsto (r, z)$. As it is also a conformal mapping, the original coordinates satisfy a Laplace equation with respect to the primed coordinates.

$$\frac{\partial^2 r}{\partial r'^2} + \frac{\partial^2 r}{\partial z'^2} = 0, \tag{13a}$$

$$\frac{\partial^2 z}{\partial r'^2} + \frac{\partial^2 z}{\partial z'^2} = 0. \tag{13b}$$

The appropriate boundary conditions follows directly from the fact that we wish to map the surface of bowl to the flat surface $z = 0$, which gives a Dirichlet condition for z on the surface. Since now the surface of the bowl is supposed to be the image of the coordinate line $z = 0$, and thanks to the fact that conformal mappings preserves angles,

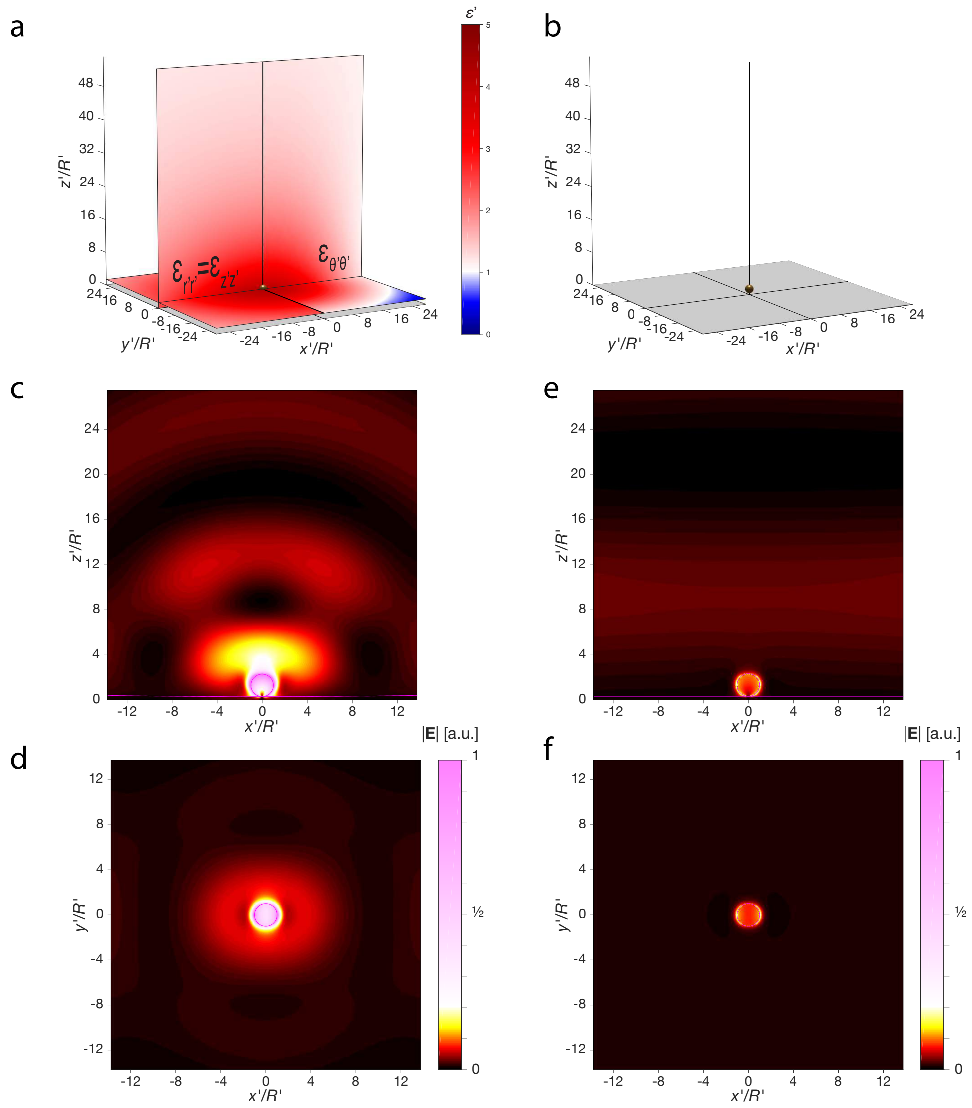

Supplementary Figure 2. **a** The spatial gradient of ϵ which can map into a surface with constant curvature. **b** The setup - a NP lies on the flat substrate with/without spatial gradient of ϵ. **c-d** The field distribution of a NP inside the ϵ gradient demonstrated in (a). **e-f** The field distribution of a NP in a homogenous media ϵ=1.

the r coordinate therefore has to satisfy a Neumann boundary condition in order to have the coordinates lines orthogonal to each others. By also consider a mapping that relaxes to identity far from the bowl (for $r \to \infty$ and $z \to \infty$) we obtain a well posed problem that can be efficiently solved numerically (we here used the finite element solver software FreeFem++ [7]). Once the resolution done, all quantities required to compute the equivalent material parameters can be deduced.

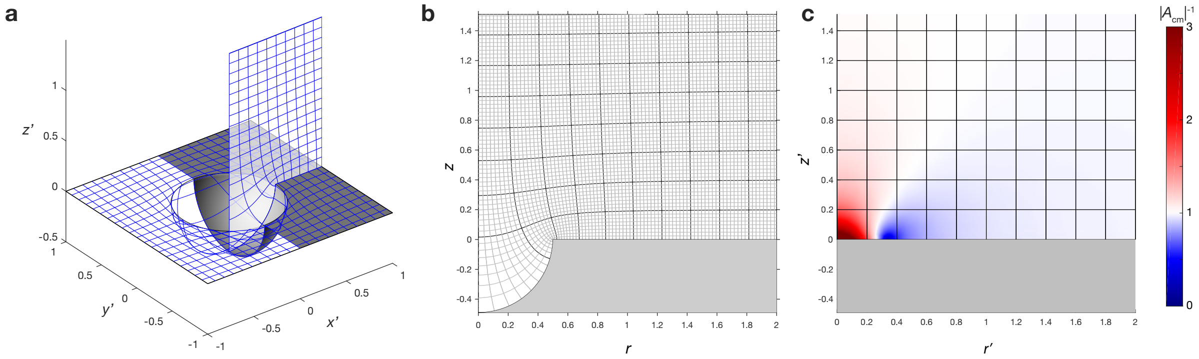

Supplementary Figure 3. Quasiconfomal mapping. **a** Projection of the virtual coordinates (x', y', z') into the physical space. **b-c** Conformal mapping basis of the whole transformation. This conformal mapping transforms the (b) geometry in the real cylindrical coordinates (r, z) into (c) a flat geometry in the virtual cylindrical coordinates (r', z'). The transformation of the material parameters relies on the inverse Jacobian of the transformation $\frac{1}{|\mathsf{A}_{\rm cm}|}$, represented with the pseudo color code.

Supplementary Note 3: Broadband feature of the curvature induced field enhancement

Supplementary Figure 4 **a-b** summarises the spatial averaged electric field $\overline{|E|}$ of NP on **a** flat and **b** warped substrate from 3D FDTD simulations. Despite the dependence of electric field on both the radius r and wavelength λ, the enhancement factor $\gamma = \overline{|E|}_{\text{curv}}/\overline{|E|}_{\text{flat}}$ is featured as broadband effect with immunity to the geometry (Figure 1f in main text). Supplementary Figure 4**c** summarises the enhancement factor of the maximum field $\gamma_{\text{max}} = E_{\text{max, curv}}/E_{\text{max, flat}}$. Prominent broadband enhancement is also observed.

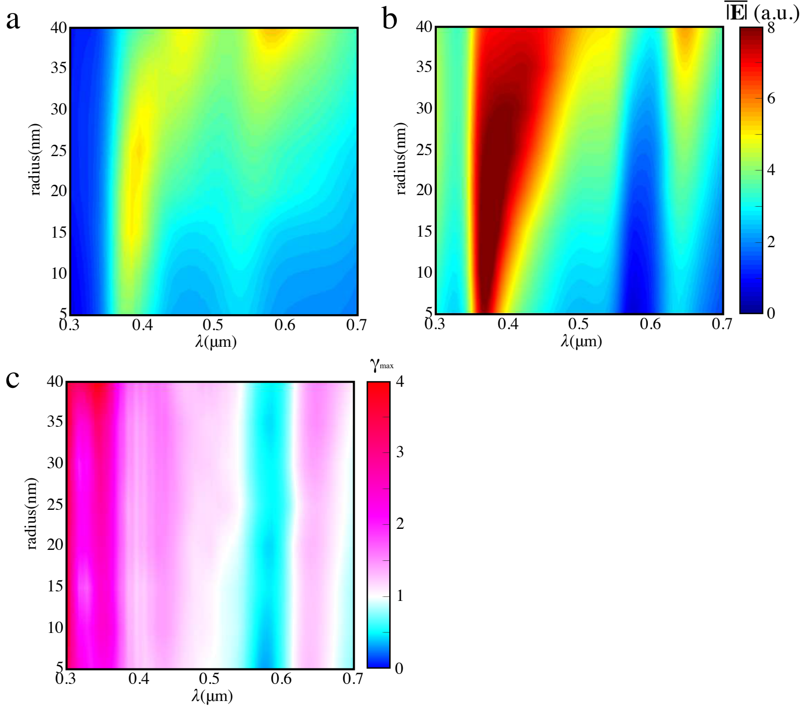

Supplementary Figure 4. The spatially averaged electric field $\overline{|E|}$ for a NP on **a** flat and **b** curved substrate for different radii and wavelengths. **c** The ratio of the maximum electric field between warped and flat substrate.

Supplementary Note 4: Quantitative analysis of the size of the hotspot

Supplementary Figure 5 summarises the results that quantitatively illustrates the enlargement of the volume of the hotspots by curved substrate. Here, we define the volume of the hotspot V_{HS} as the region in air with magnitude of the electric field greater than a threshold value $|E|> E_c$. Examples are provided to illustrate the hotspots in Supplementary Figure 5a-b for a flat substrate and Supplementary Figure 5c-d for a curved substrate, with the region of $|E|< E_c = 5$ not plotted. A clear enhancement of the hotspot size at curved case is observed from the two examples. We calculate the value of V_{HS} at different wavelengths with different nanoparticle sizes from 3D FDTD simulations, with results shown in Supplementary Figure 5e-f. The hotspot volume is normalised to the volume of the nanoparticle V_{NP}. In the case of curved substrate (Supplementary Figure 5e), the hotspot size is unambiguously enlarged at different wavelengths, with the size even greater than the nanoparticle.

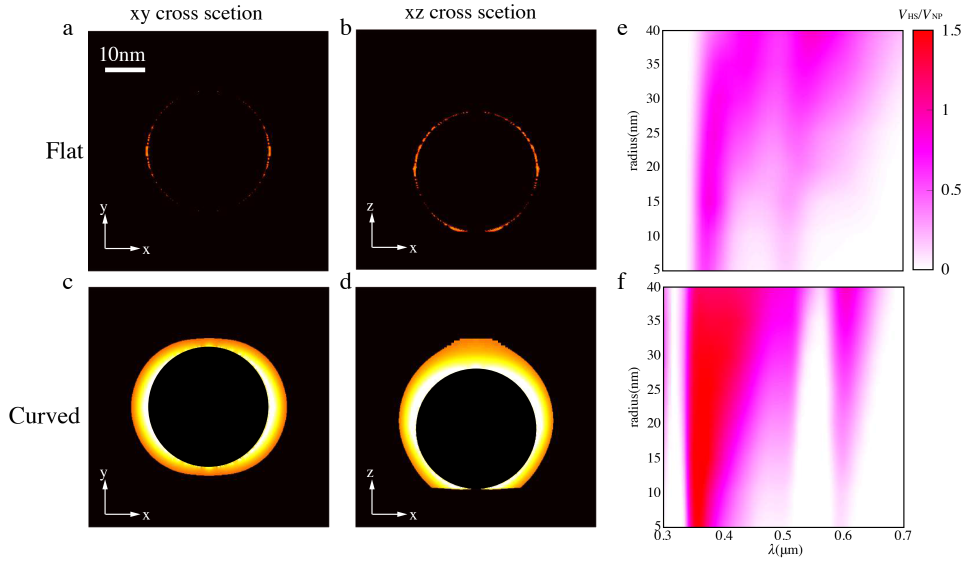

Supplementary Figure 5. **a-d** Demonstration of the hotspot area for single NP on (a-b) a flat substrate and (c-d) a curved substrate at 345 nm. Only the region in air with $|E|> E_c = 5$ is illustrated. **e-f** Hotspot volume at different wavelengths and NP radii for single NP on (e) a flat substrate and (f) a curved substrate. The hotspot volume V_{HS} is normalised to the volume of the nanoparticle $V_{NP} = \frac{4}{3}\pi r^3$.

Supplementary Note 5: Spacial dependence of the curvature induced field enhancement

Here, we investigate the spatial variation of the enhanced field when the nanoparticle is located at different positions on the warped substrate based on 3D full-wave simulations. Supplementary Figure 6**a** demonstrate the configuration of the simulation, where the position is determined by the inscribed angle θ. In Supplementary Figure 6**b**, we summarise the simulation results of a single NP with $r = 30$nm at different positions inside the arc at different wavelengths. All the values are normalised to the case with NP in the centre ($\theta = 0^o$) with a parameter defined as $\gamma_{angle} = \overline{|E(\theta)|}/\overline{|E(0^o)|}$. In addition, a comparison with flat substrate is presented in Supplementary Figure 6**c**, where all averaged field is normalised to the case in NP on a flat substrate, $\gamma_{comp} = \overline{|E(\theta)|}/\overline{|E_{flat}|}$

Below 500 nm, a prominent decrement in the field intensity is observed as the NP deviates from the centre (increment of θ). Quite interestingly, between 500 to 600 nm, there is an enhancement peak around $\theta = 30^o$. Inside this region, the light energy is confined at the middle of the arc, which corresponding to $\theta = 30^o$ with better enhancement. Supplementary Figure 6d-e illustrate the field distribution for different θ at 345nm and 550nm, providing more details about the two different situations mentioned above.

In Fig. 1f of the main text, there is a degradation of γ around 550nm, matching the first row of Supplementary Figure 6d-e when $\theta = 0^o$. The enhancement around 550 nm as the increment of θ is able to compensate the degradation when the surface is covered with multiple particles. As a result, the improvement of the Raman intensity for pump at 514nm still exist, as proved by the experimental result ($\alpha_I > 1$) in Fig.3 of the main text. To make more rigorous evaluation, we further calculate the value of $\int_{\mathcal{S}} {E_s}^4 ds$, considering the Raman signal is proportional to the fourth power of the electric field E_s at the surface area $\mathcal{S}$. And the enhancement from a flat to curved substrate is demonstrated in Supplementary Figure 7a at different angle θ. The enhancement from 10° to 50 ^{o} can compensates the degradation at 514nm. Supplementary Figure 7b provide the averaged enhancement through the angle with single nanoparticle. Due to the limit of computational resources currently available, we considered single particle simulations averaged at different positions. These simulations qualitatively match the experimental results and reproduce the main features of our samples. Using position-averaged value of a single nanoparticle instead of the curved substrate fully covered with nanoparticles cause the discrepancy between the simulation and experimental results.

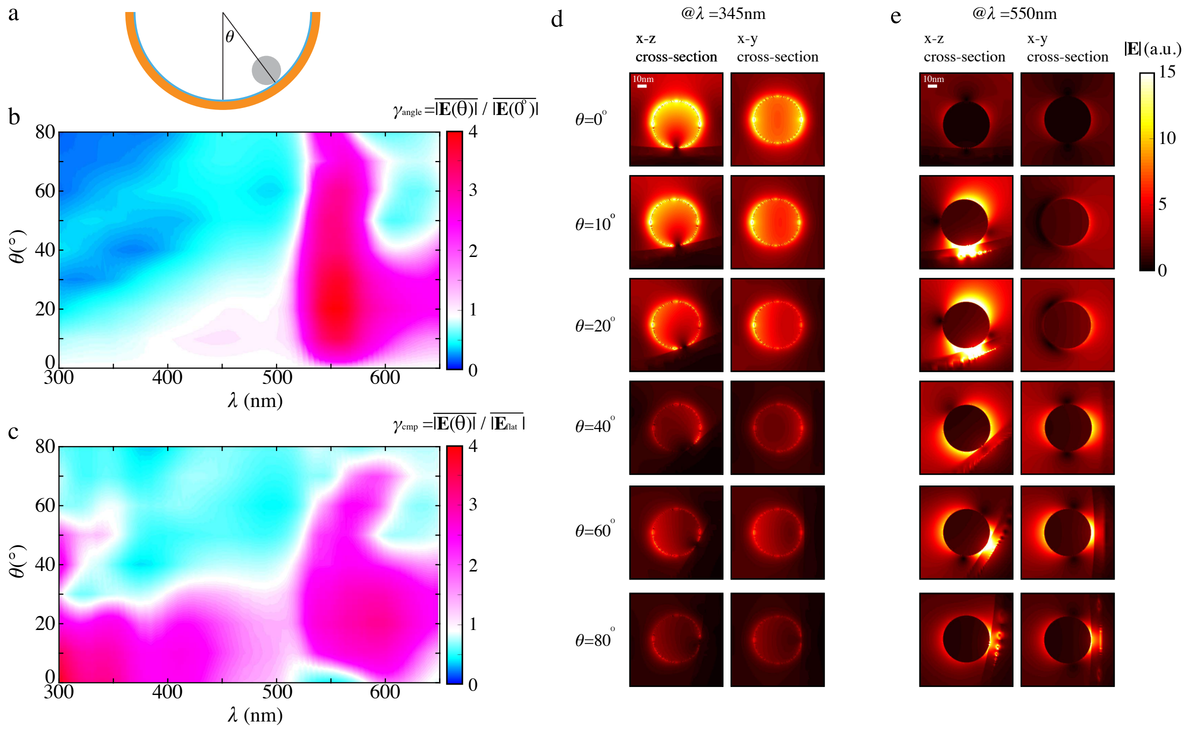

Supplementary Figure 6. The spatial dependence of the field enhancement for a single NP on the warped substrate. **a** The configuration of the simulation, with inscribed angle θ a parameter representing the different positions on the curved substrate. **b** The angle-dependent field variation for a NP with r=30nm at different wavelengths. The value is normalised to a single NP on the bottom ($\theta = 0^o$). **c** The angle-dependent field enhancement for a NP with r=30nm at different wavelengths compared with a NP on a flat substrate. **d** Field distributions of the NP at $\lambda = 345$nm for $\theta = 0^o$, 10°, 20°, 40°, 60° and 80°. **e** Field distributions of the NP at $\lambda = 550$nm for $\theta = 0^o$, 10°, 20°, 40°, 60° and 80°.

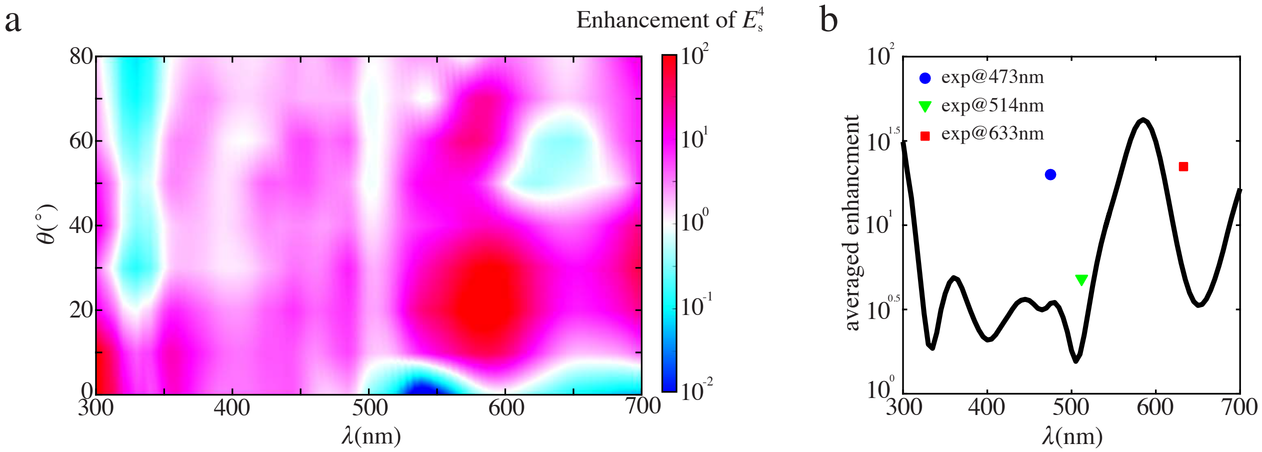

Supplementary Figure 7. (a)The enhancement of E^4 from flat to curved substrate for different wavelengths and θ. (b) The angle-averaged enhancement. Experimental data is also listed as a reference.

Supplementary Note 6: enhancement of hotspot formed by inter-nanoparticle coupling

In the main text, we investigate the field enhancement of a single nanoparticle on warped/flat substrate, while in our experimental realisation, there were multiple nanoparticles on a warped substrate. In this section, we investigate the impact of the curvature to the hotspots formed by inter-particle coupling, an indispensable factor to enhance the hotspot density in the SERS system.

For simplicity but not loss of generality, we focus on the case of two nanoparticles sitting on the flat/warped substrates, as illustrated in Supplementary Figure 8a. The distance d between the NPs controls the coupling strength. For a pair of NPs sitting on a flat substrate, hotspot can be formed between the particles, as illustrated in Supplementary Figure 8b with different values of d. Similar to the single particle case, the curvature induced gradient of the refractive index improves the light confinement, generating brighter and larger hotspots, as demonstrated in Supplementary Figure 8c. For a quantitative comparison as we did for the single NP case, here we define an enhancement factor $\gamma_{twin} = \overline{|E_l|}_{warp}/\overline{|E_l|}_{flat}$, where $|E_l|$ the amplitude of the electric field along the line between two centres of the nanoparticles, as illustrated in Supplementary Figure 8d. Supplementary Figure 8e summarises the enhancement factor γ_{twin} at different wavelengths and coupling strength. A broadband enhancement is observed at different d, which is quite similar to the single NP case (Fig.1f in the main text), demonstrating the generality of the curvature induced enhancement that is insensitive to the inter-particle coupling. The degradation around 550nm is due to the localisation for the light in the middle of the nanobowl, similar to the single NP case.

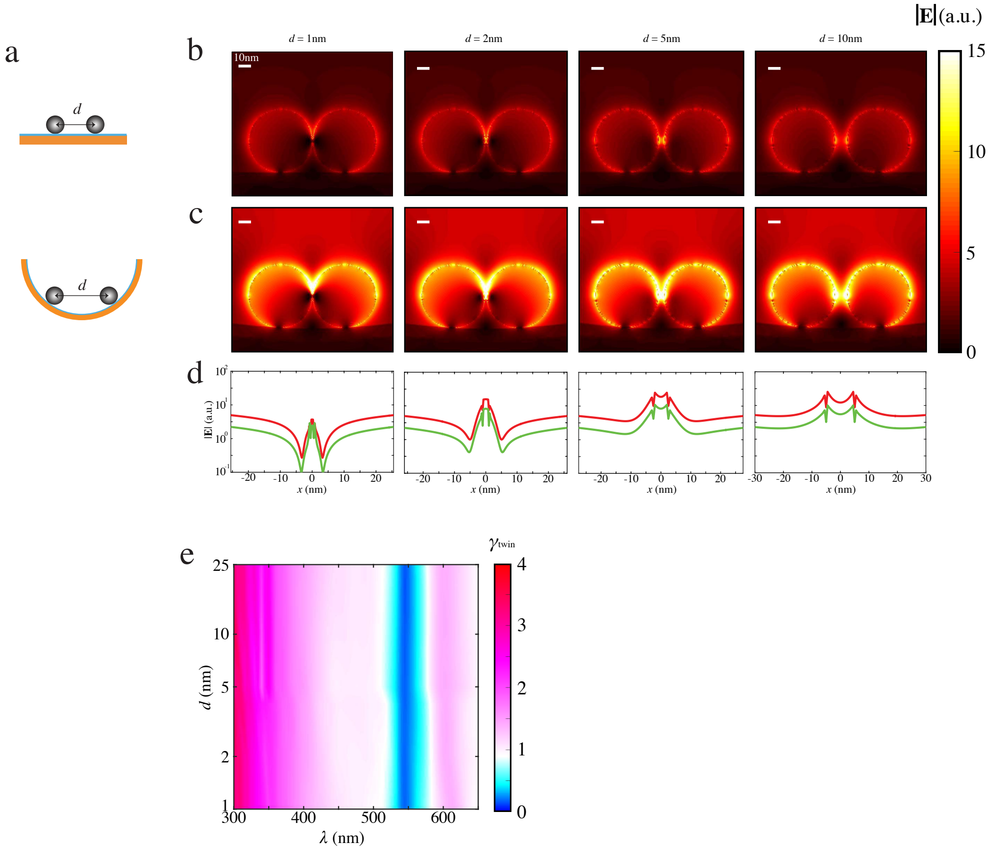

Supplementary Figure 8. The field enhancement with twin NPs with varying inter-particle coupling. **a** The configuration of the simulation, with two NPs on a flat or warped substrate. **b** Field distributions of the twin NPs on a flat substrate at $\lambda = 345$nm for d = 1,2,5,10nm. The scale bar represents 10nm. **c** Field distributions of the twin NPs on a warped substrate at $\lambda = 345$nm for d = 1,2,5,10nm. **d** The magnitude of the corresponding electric fields along the line between two centres of the twin NPs. **e** Enhancement factor γ_{twin} for different distance d and wavelength λ.

Supplementary Note 7: Preparation details of Au nanobowl array

The monodisperse silica nanospheres (size dispersion 1.9 %) used in the work were purchased from Duke Scientific Corps. The 2D silica colloidal crystals (CCs) were prepared by injecting an aqueous solution of colloidal dispersion with a suitable concentration into a channel that was formed from two parallel quartz slides separated by a U-shaped spacer. The quartz slides had been pretreated to render their surface hydrophilic by soaking in a solution of 30 % hydrogen peroxide at 80 ^{o}C for 30 min. After drying in air, highly ordered CCs were grown within the channel under capillary force. The prepared 2D CCs acted as a periodical template and gold was then evaporated on the top of the nanospheres in a vacuum of 10^{-4} Pa at a rate of 1Å/s to the desired thickness by an home-made thermal evaporation coating system. Then the silica template was etched by using hydrogen fluoride acid to leave the Au nanobowls in the solution. The upward interconnected Au nanobowl arrays can be prepared via a transferring process, the schematic of the fabrication steps were shown in Supplementary Figure 9. Supplementary Figure 10 **a** and **b** show the SEM images of the metallic periodic structure before dissolution of the silica template. Supplementary Figure 10**c** shows the SEM image of Au nanobowl array supported on clean quartz substrate.

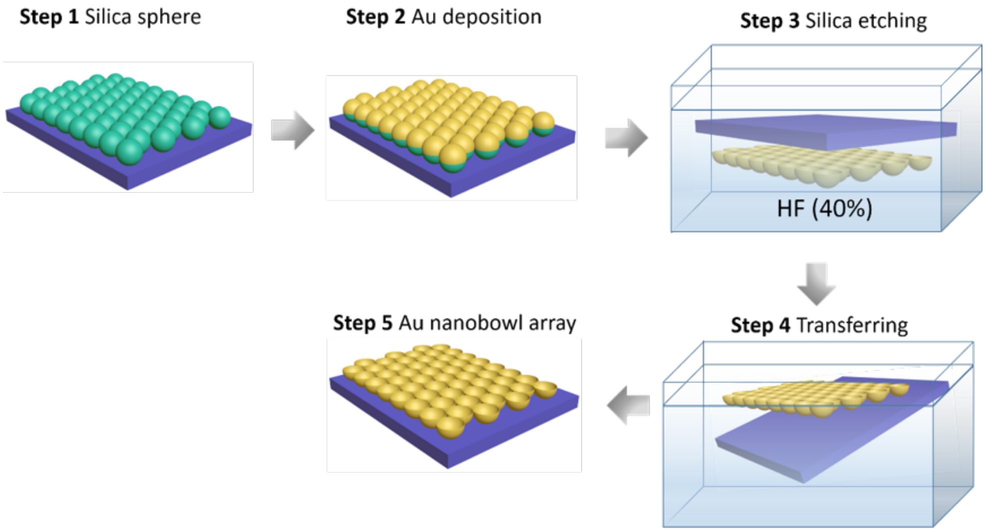

Supplementary Figure 9. Schematics of the fabrication procedure of the Au nanobowl array. Step 1: self-assembly of silica nanosphere layer; step 2: Au deposition; step 3: silica etching (HF 40%); step 4: transforming; step 5: Au nanobowl array.

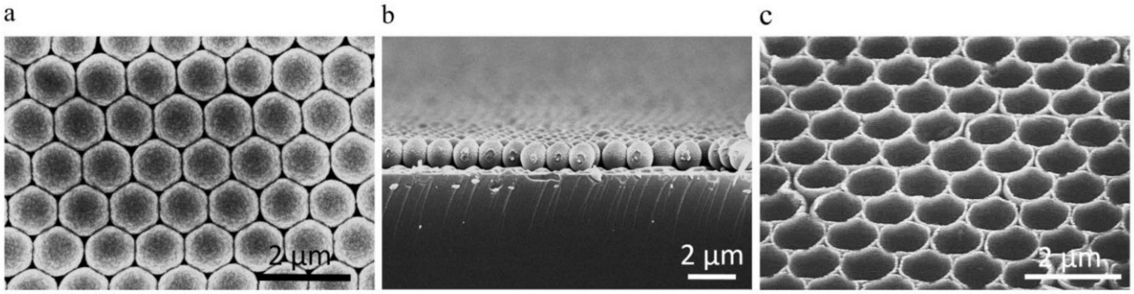

Supplementary Figure 10. **a** SEM image of SiO_2/Au nanoshpere array. **b** SEM cross-sectional image of SiO2/Au nanosphere array. **c** SEM images of the hexagonal close-packed Au nanobowl array.

Supplementary Note 8: Cluster beam deposition system

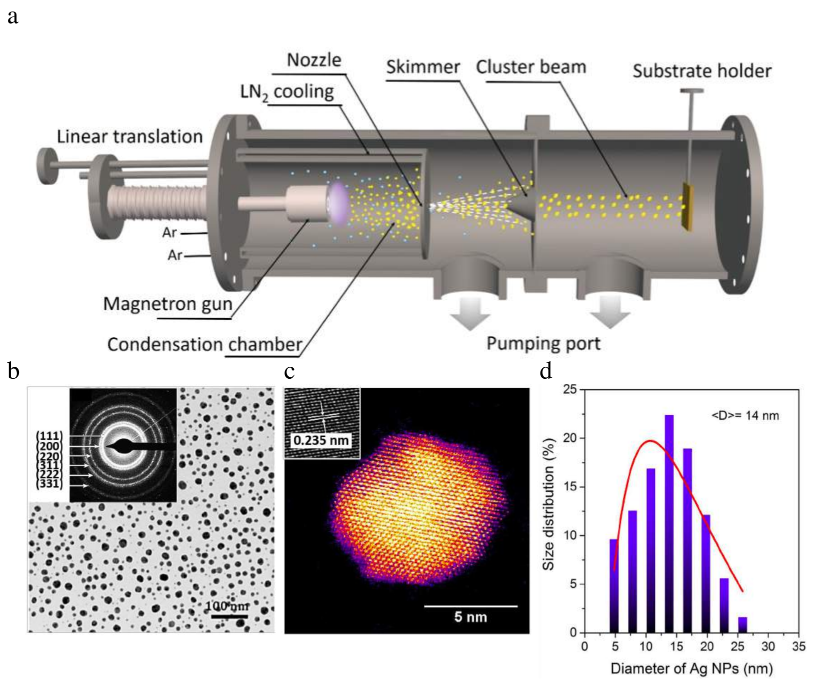

Supplementary Figure 11. **a** Schematic diagram of a magnetron plasma gas aggregation cluster beam deposition process. **b** BF-STEM image of as-deposited Ag NPs. The inset shows the selected-area electron diffraction (SAED) pattern of Ag NPs. **c** Typical HAADF-STEM image a single Ag NP.The inset is BF-STEM image of Ag NP and the corresponding lattice constant has been marked in the figure. **d** Size distribution of as-deposited Ag NPs. The average size of as-deposited Ag NPs is about 14 nm.

Gas phase cluster beam deposition process was used to deposit Ag NPs on the inner wall surface of Au/SiO_2 NBC structure. In this fabrication, Ag NPs were generated in a magnetron plasma gas aggregation cluster source and deposited on substrates directly, as shown in Supplementary Figure 11a. The deposition was performed in a high-vacuum chamber equipped with the cluster source. A silver plate (50mm*3mm) with high purity (99.99%) was used as the sputtering target. A DC power supply was used for the sputtering of Ag target in argon gas (purity, 99.99%) ambient with a pressure of ∼100 Pa, maintained by passing argon gas to the liquid nitrogen-cooled aggregation tube. A stable magnetron discharge ran with input power of 40 W. Pure Ag NPs were initially formed through a supersaturated vapour of metal atoms by sputtering of the target under argon atmosphere. The nanoparticles were swept by the gas stream out of the aggregation tube into vacuum through a diaphragm, where the nanoparticles growth was effectively stopped. The nanoparticles continued to pass through a skimmer (the second diaphragm)

into a high vacuum (10^{-4} Pa) chamber and forming a collimated nanoparticle beam with a high speed of ∼1000 m/s, and then deposited on the surface of substrates. The deposition was carried out at a rate of 0.5 Å/s for 10 min. In situ annealing was carried out for 10 min at 150 ^{o}C. The cluster beam deposition rate was monitored by a quartz crystal microbalance (QCM). The number and number density of the nanoparticle layer could be adjusted as desired by controlling the deposition time and cluster beam flux rate, since the cluster beam could be instantly cut off by a shutter to make a careful control of the deposition mass. The annealing converts the as-deposited small size Ag NPs into larger Ag NPs due to Ostwald ripening.

The scanning transmission electron microscopy (STEM) investigation was performed using a JEOL instrument (JEM2100F) with a spherical-aberration corrector (CEOSGmbH). The images were acquired using high-angle annular dark field (HAADF) and bright field (BF) detectors. For facilitation of the STEM observation, Ag NPs were deposited on SiO_2 films supported by copper grids. Further details of the composition of NP-on-WS structure were provided by energy-dispersive X-ray spectra (EDX) elements mapping analysis.

Supplementary Figure 11b shows the BF-STEM image and and selected-area electron diffraction (SAED) pattern of as-deposited Ag NPs. According to the selected-area electron diffraction (SAED) pattern, it can be deduced that the Ag NPs were in a crystalline state. The main diffraction rings can be assigned to the Ag FCC phase corresponding to the (111), (200), (220), (311), (222) and (331)crystal planes. Supplementary Figure 11c shows the HAADF-STEM and BF-STEM images of a single Ag NP. The lattice fringes have an interplanar spacing of 0.235 nm, corresponding to the (111) planes of the face-centered cubic (FCC) structure of metallic Ag, which clearly reveals the crystalline nature of the Ag NPs synthesized in the present study. Supplementary Figure 11d shows the size distribution of as-deposited Ag NPs, the average size of as-deposited Ag NPs is about 14 nm.

Supplementary Figure 12 a-d show the optical photographs and SEM images of the three large area samples. Elemental mapping analysis was also performed to verify the spatial distribution of the different elemental compositions in individual NP-on-WS nanostructure. As shown in Supplementary Figure 12e, the Ag and Au elements can be clearly identified in the wrapped space.

Supplementary Figure 13 illustrates the size statistics of the nanoparticles used for NP-on-WS/NP-on-FS. We can find that the distribution of the diameter of NPs is in line with the logarithmic normal distribution, and the mean sizes of Ag nanoparticles are about 48 nm.

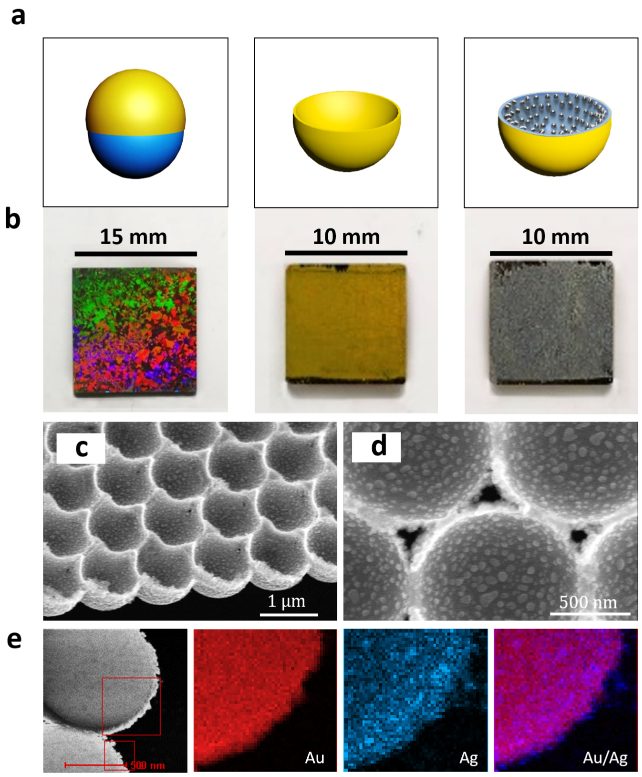

Supplementary Figure 12. **a** Schematic illustration and **b** optical photographs of the three nanostructures: SiO_2/Au nanoshpere array, hexagonal close-packed Au nanobowl array and NP-on-WS structure. **c** Low magnification SEM image of the NP-on-WS structure. **d** High magnification SEM image of the NP-on-WS structure. **e** HAADF-STEM image (left) and elemental mapping results for the boxed area in the main image of NP-on-WS structure.

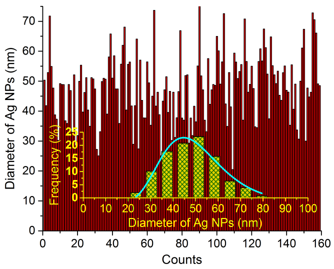

Supplementary Figure 13. Size histogram of the Ag NPs in the NP-on-WS/NP-on-FS structure and corresponding Log-Normal fits.

Supplementary Note 9: More details of the boardband enhancement experiment

For the clarification of the contribution from warped substrate, we also fabricate substrates with nanobowl array only, as illustrated in Supplementary Figure 14. Even without Ag NPs, the Raman scattering from substate with NBs (blue line) is remarkably boosted compared with that of bare gold film given by the baseline (black line), by the virtue of the gradient of n_{eff} that strongly localises light around the molecules.

To demonstrate the broadband response and the curved substrate, we make the measurement at a fourth wavelength at 785 nm, with results summarised in Supplementary Figure 15. Despite the reduction of the Raman signal at 1360 cm^{-1} resulted from the deviation of R6G absorption band (Supplementary Figure 15a), α_I remains the similar value (> 20) owing to the broadband feature of the curved substrate.

There is a degradation of the field enhancement between our experimental data compared with the simulations, which is owing to fabrication imperfection and enhancement reduction for NPs deviating from the centre of the nanobowl.

The experimental conditions including the laser pump power (used to normalise the Raman intensity) and the analyte concentrations for different structures are listed in the following table.

	laser power(mW)	dye concentration (M)
NP-on-WS	0.01	10^{-6}
NP-on-FS	0.01	10^{-6}
NB array	0.1	10^{-4}
Au film	1	10^{-2}

Supplementary Table 1. SERS experimental conditions

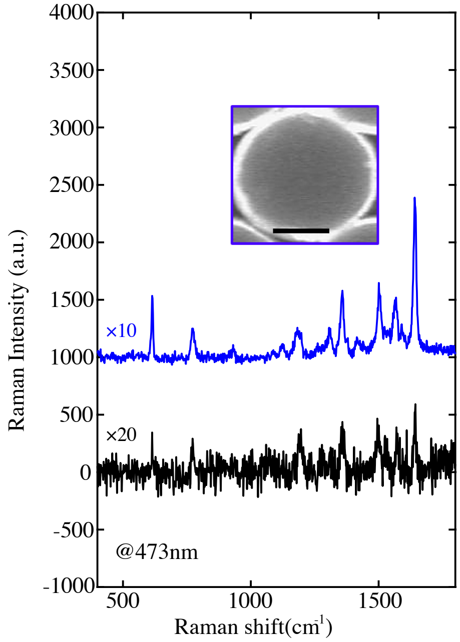

Supplementary Figure 14. The typical SERS results for bare nanobowl array. A prominent boost induced by curvature is illustrated even without NPs.

a

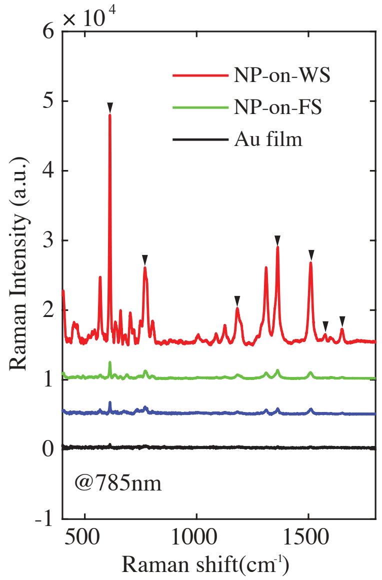

b

	473nm	514nm	633nm	785nm
$\overline{EF}$@1360cm^{-1} (NP-on-WS)	1.11 ×10^9	1.20 ×10^9	0.54 ×10^9	1.16 ×10^9
$\overline{EF}$@1360cm^{-1} (NP-on-FS)	0.54 ×10^8	2.47 ×10^8	0.24 ×10^8	0.50 ×10^8
α_I ($I_{NP\text{-}on\text{-}WS}/I_{NP\text{-}on\text{-}FS}$)	20.4	4.8	22.2	23.2

Supplementary Figure 15. SERS measurement with 785nm laser. **a** SERS signals of NP-on-WS, NP-on-FS and the Au film. **b** Calculated $\overline{EF}$ and α_I. The values of other three wavelengths are included as a reference.

Supplementary Note 10: Bi-Analyte single molecule SERS base on modified principal component analysis

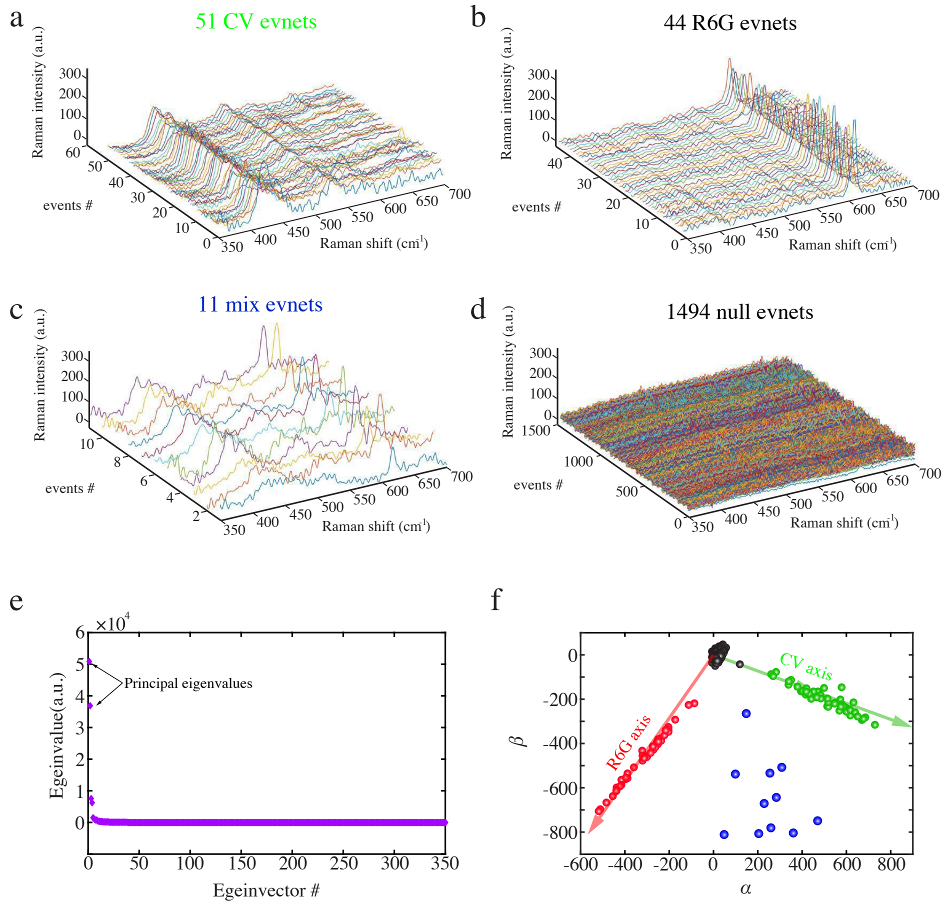

Supplementary Figure 16. **a** Raman spectra of 51 CV events. **b** Raman spectra of 44 R6G events. **c** Raman spectra of 11 mixed events of CV and R6G. **d** Raman spectra of 1494 null events. **e** Eigenvalues (in decreasing order) obtained from MPCA analysis of 1600 spectra with 2 principal eigenvalues. **f** Coefficient plot of (α', β') without orthogonalisation

We verify the single molecule sensitivity based on modified principal component analysis [3]. We obtain 400 spectra from the 2D Raman scanning on the substrate, as illustrated in Supplementary Figure 16 a-d. The spectra is composed of 51 events of single CV molecule scattering, 44 events of single R6G molecule scattering, 11 mixed events and 294 null event without any active molecules. Then, we followed the modified principle component analysis method developed by Etchegoin et. al. to statistically prove

single-molecule sensitivity with the following steps,

- Discretize all the 1600 spectra, putting them into a matrix by T rows and N columns, with where T and N are the number of spectra and wavelengths, respectively..
- Subtract each row of the matrix with mean intensity, $\hat{M} = M - \overline{M}$.
- Calculate the covariance of the matrix $\hat{M}$, $V = cov(\hat{M})$
- Calculate the eigenvectors and eigenvalues of V. The eigenvalue of the matrix is plotted in Supplementary Figure 16e, the first two eigenvectors dominant, corresponding to the statistical behaviour of the bi-analyte method for single molecule detection.
- Calculate the coefficient matrix $C' = \binom{\alpha'}{\beta'}$ by a matrix operation equivalent to scalar product of spectra with first two eigenvectors. The coefficient is plotted in Supplementary Figure 16f, unambiguously demonstrating the different classes corresponding to R6G, CV, mixed and null events.
- Apply a linear transformation to make the two principal eigenvector orthogonal, with corresponding coefficients $C = \binom{\alpha}{\beta}$ plotted in the Fig. 4c in the main text.
- Calculate the histogram (Fig. 4d in the main text) that represents the relative contribution of the number of molecules to the total signal by $p = 1/(1 + \beta/\alpha)$.

We also demonstrate the SERS spectra of CV as a reference shown in Supplementary Figure 17.

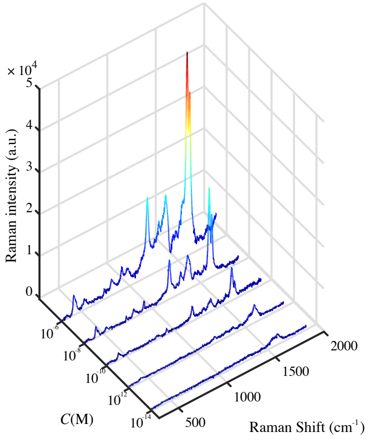

Supplementary Figure 17. SERS spectra of CV at 5 different concentrations.

Supplementary Note 11: Uniformity and reproducibility of the SERS substrate at large scale

We perform Raman mapping for the investigation of the uniformity for our SERS systems, as illustrated in Supplementary Figure 18. Taking the advantage of beam cluster deposition with large-scale producibility, we are able to prepare a SERS substrate with size of 1 cm, as illustrated in Supplementary Figure 18a. We select two arbitrary regions on the sample for the Raman mapping measurement, as summarised in Supplementary Figure 18 b-e. The step is chosen as 0.5 μm per point and the dwell time as 1s for sample scanning. Supplementary Figure 18 b and d are the corresponding 2D mappings at concentration of 10^{-6}M, with the brightness of the grid representing the signal intensity at 1360 cm^{-1}. Under this high concentration, the spatial dependence of the enhancement (shown in Section IV) is averaged out, owing to the comparable sizes of laser beam (about 700nm) and the NB (1μm). The discernible fringes in the 2D mapping corresponds to edges of the NB where curvature-induced enhancement does not occurs. Supplementary Figure 18 c and e illustrate the peak value along the dashed line in Supplementary Figure 18 b and d respectively, with a relative standard deviation (RSD) of 6.78% and 5.73%. Repeatable and stable spectra unambiguously reveal good uniformity and reproducibility of our substrate as result of periodic Au NBs and evenly distributed Ag NPs, comparable with the results in ref[34,41,42] in the main text. Furthermore, the dense hotspots with enlarged volume can allow more molecules to be activated and probed within the detection volume and, as a consequence, mitigate the effect due to local structure variation and improve the signal uniformity.

When the concentration of the dye is reduced, the fluctuations of the intensity is due to the inhomogeneous enhancement (as discussed in section IV) becomes more signifcant, resulting in an increased RSD of 14.08% with concentration of 10^{-12}M. Such value is calculated from Fig.4a in the main text, excluding the none events.

In addition to NP-on-WS substrate, we also investigate the uniformity of the substrate with NBs only, as shown in Supplementary Figure 19 a-b. Excellent uniformity is observed for the system, with relative standard deviation (RSD) of 5.92% for the peaks at 1360 cm^{-1}. Regarding the RSD of 6.82% for NP-on-WS substrate, the minute increment of non-uniformity is resulted from the introduction of additional NPs. The uniformity of both NP-on-FS and NB array also demonstrate homogenous distribution of the dye (R6G) with concentration of 10^{-6}M.

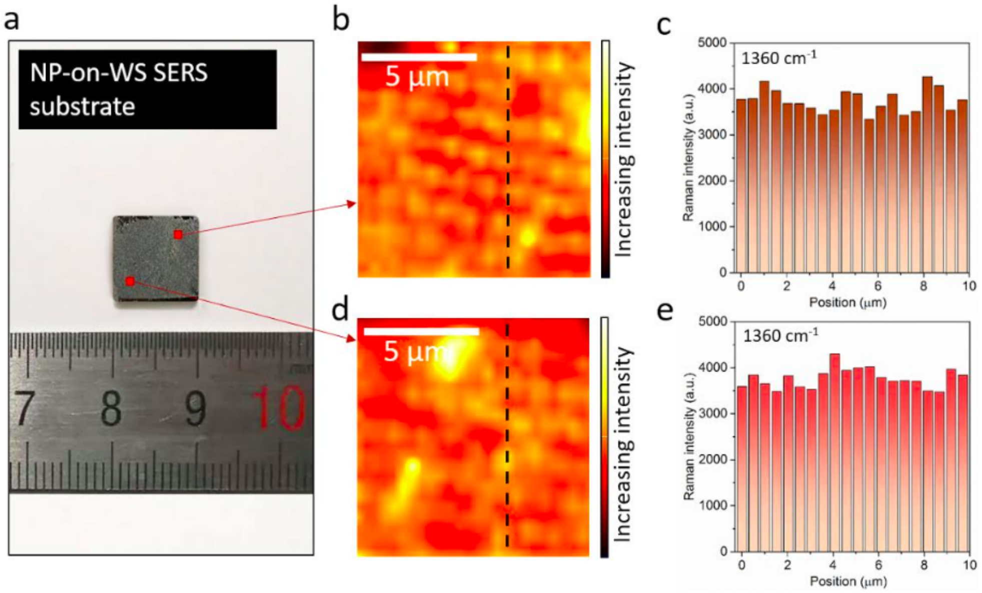

Supplementary Figure 18. High quality of NP-on-WS SERS system with good uniformity and reproducibility at large scale. **a** Picture of the SERS substrate with size around 1cm. **b** Raman spectra mapping of one arbitrarily selected region for the peak intensity at 1360 cm^{-1}. **c** The corresponding histogram of peak intensity values along the dashed line of (b). **d** Raman spectra mapping of the other arbitrarily selected region for the peak intensity at 1360 cm^{-1}. **e** The corresponding histogram of peak intensity values along the dashed line of (d). The concentration of R6G concentration is 10^{-6}M.

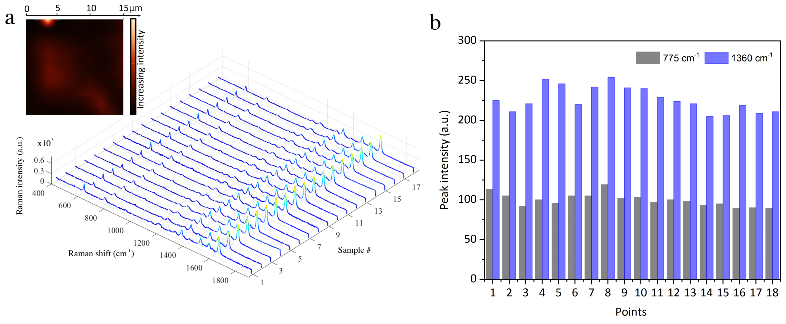

Supplementary Figure 19. **a** Raman spectra of 10^{-6} M R6G solution acquired from 18 random spots along the diagonal line of mapping area on the Au NB array. The inset shows the intensity spatial distribution of the R6G 1360 cm^{-1} peak over the mapping area obtained from the R6G-decorated Au NB only. **b** The corresponding histograms of SERS spectral intensities along the diagonal line of mapping area on Au NB only.

Supplementary Note 12: Impact of the nanoparticle density on SERS signals

Here in this section, we investigate the SERS substrates with different nanoparticle coverage rates, in order to experimentally demonstrate the role that the curvature plays under different coupling strengths predicted by simulations in Section V.

Supplementary Figure 20a illustrates NP-on-WS structures with four configurations of different nanoparticle coverage rates. The corresponding Raman spectra are measured and shown in Supplementary Figure 20b. The increase in density provides more hotspots and shortens the distance between the NPs, consequently boosting the inter-particle coupling and producing larger hotspot region for enhanced Raman peaks. Similar phenomena are also observed for the NP-on-FS structures, as summarised in Supplementary Figure 20 c-d. The cluster beam technique can readily produce similar number of nanoparticles on flat/curved substate with fixed surface area, insuring the averaged NP distance at the same level. Supplementary Figure 20e quantitatively illustrates the Raman intensity peaks at 1360 cm^{-1} for the two structures at different coverage rates. Despite the variation of the peak intensity, the enhancement from the flat to warped substrate $\alpha_I = I_{\text{NP-on-WS}}/I_{\text{NP-on-FS}}$ remains almost the same (~ 20), demonstrating the generality of the improvement from the curved substrate.

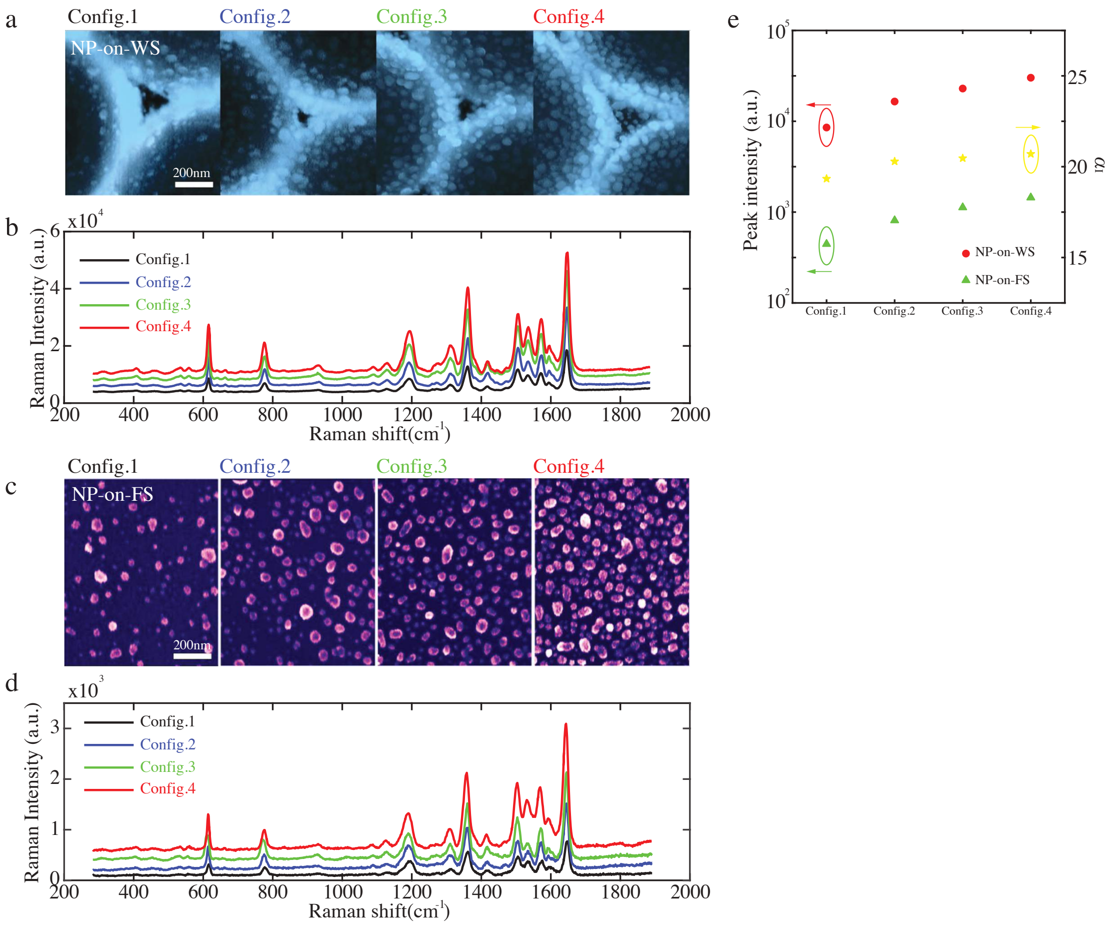

Supplementary Figure 20. **a** SEM images of NP-on-WS structures with four different nanoparticle coverage rates Config.1-4. **b** The corresponding Raman signals for the samples in (a). **c** SEM images of NP-on-FS structures with four different nanoparticle coverage rates, which match the density in (a). **d** The corresponding Raman signals for the samples in (c). **e** A comparison of the peak intensity at 1360 cm^{-1} for flat and warped substrates and α_I for different nanoparticle density. The laser pump is at 473nm.

Supplementary Note 13: Reflection measurement for the periodic SERS structures

Supplementary Figure 21 demonstrates the reflection spectra for three different structures: Au film, periodic nanobowls and NP-on-WS. Despite the periodic configuration of the nano-bowls, the reflection spectrum does not show any sharp spectral features that are usually associated with photonic band structure, demonstrating the negligible role of the Bragg reflection for the hotspots formation.

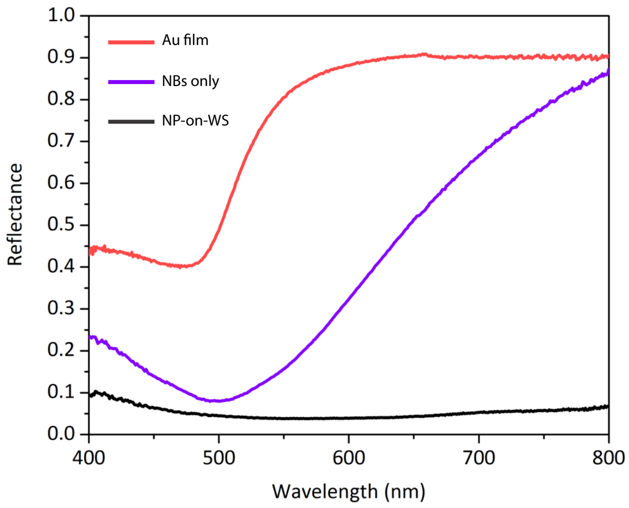

Supplementary Figure 21. Reflection spectra of 3 different structures: Au film, periodic nanobowls and NP-on-WS.

Supplementary Note 14: Enhancement factor estimation based on the monolayer analyte coverage assumption

In the main text, we calculate the EF based on a global averaged one $\overline{EF}$ instead of the EF with monolayer coverage assumption (with sample rinsing process) for the following reasons:

- The EF can be directly calculated from the experimental measurement while the other one needs prior parameter estimation such as the nanostructure density and surface area, molecule cross-section.
- For practical applications, determination of the concentration of an analyte (as we did in Fig.3g in the main text) is as important as identifying the analyte. The rinsing process may impact the accuracy of the concentration test due to the removal of the extra molecules.
- The rinsing process removes molecules and consequently reduce the Raman signal (sensitivity). Such extra step may not be needed for practical applications.

Meanwhile, it is also meaningful to calculate the EF with estimation of monolayer analyte coverage assumption, for the comparison with the averaged one and other references.

The most widely used definition for the SERS EF is defined as

$$EF = \frac{I_{\text{SERS}}/N_{\text{Surf}}}{I_{\text{Ref}}/N_{\text{Vol}}} \tag{14}$$

where N_{Vol} is the number of molecules in the scattering volume for the Raman (non-SERS) measurement and N_{Surf} is the average number of adsorbed molecules in the scattering volume for the SERS experiments. When the SERS substrate is covered by a monolayer of analyte, the enhancement factor can be calculated as the following [12]:

$$EF = \frac{I_{SERS}/(\mu_M \mu_S A_M)}{I_{\text{Ref}}/c_{\text{Ref}} H_{\text{eff}}} \tag{15}$$

where I_{SERS} and I_{Ref} are the intensity from SERS substrate and reference after normalisation to the pump laser power, c_{Ref} is the concentration of the solution used for the non-SERS measurement, H_{eff} is the effective height of the scattering volume, A_M represents the surface area of the metallic structure, μ_M the surface density of the individual nanostructures producing the enhancement, and μ_S is the surface density of molecules on the metal.

According to our configurations, we set $A_M = \pi r^2 = 7.85 \cdot 10^{-17} m^2$ (in reality, the whole sphere cannot be achieved at the contact between NP and curved substrate), $\mu_S =$

$0.059 \cdot 10^6 \mu m^{-2}$ for R6G [9], $c_{\text{Ref}} = 10^{-2} M$ and $H_{\text{eff}} = 80 \mu m$ [8, 5]. The density of the NPs of NP-on-WS and NP-on-FS is measured by the SEM (more details shown in the following section).

For this definition of EF, a monolayer coverage of analyte assumption is required. Therefore, we rinse the sample in ethanol to remove excess molecules that are not covalently bound to the metal surface and gently blow the substrate dry with nitrogen gas. To test the rinsing process can effective remove molecules that are not chemically absorbed by the nanostrucutres, we measure the Raman signals with different dye concentrations from 10^{-5}M to $4 \cdot 10^{-5}$M, as shown in Supplementary Figure 22a. Despite the difference in the concentration, the Raman intensity remains nearly the same (Supplementary Figure 22b), implying the remove of extra molecules on the NPs.

We implement the same experiment in the main text for different pump lasers, with result summarised in Fig.S23a-e. Similar spectra are observed compared to the sample without rinsing shown in Fig.3 in the main text and Fig.S15a. The peak intensity is reduced due to removal of the extra molecules, with a quantitative analysis in Fig.S 24a. We define the parameter $\eta = I_{\text{with rinsing}} / I_{\text{w/o rinsing}}$, the ratio between unwashed and washed Raman signal. Due to the fast soaking time for only 60s, the signal does not experiences a big degradation. Supplementary Figure 23e summarises the EF at different wavelength for both NP-on-WS and NP-on-FS. Compared to the averaged $\overline{EF}$ in Fig.3e in the main text, EF based on single layer coverage assumption has a large value. This can be explained by the fact that the monolayer assumption only counts the "best" molecules with direct contact(chemically absorbed) but neglects the molecules (by rinsing them away) that does not directly contact with the nanostructure while the global averaged one taking account for all the molecules on the testing substrate. However, the curvature-induced enhancement parameter α_I remains almost the same (Supplementary Figure 24b), unambiguously demonstrating intensity enhancement by the curvature from a flat to a warped substrate.

The SERS measurement with different soaking time is also implement with rinsing process, as shown in Fig.S23f-g. Similar spectra are observed as the case without rinsing.

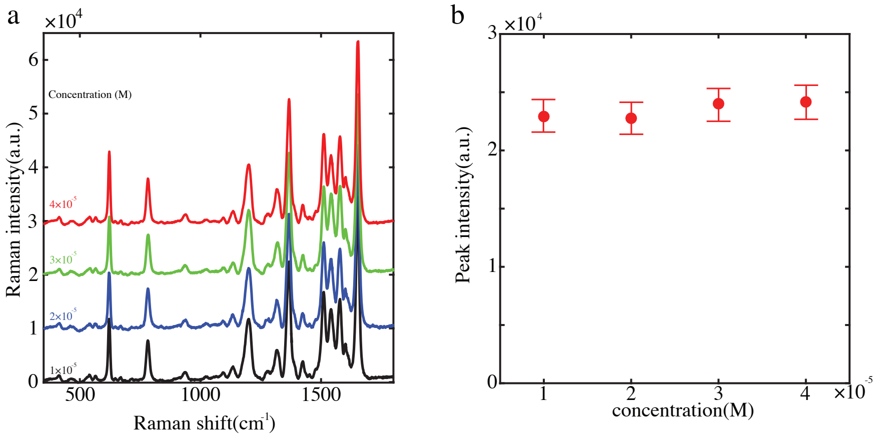

Supplementary Figure 22. **a** SERS spectra from rinsed sample with different concentrations. **b** The corresponding peak intensities at 1360 cm^{-1}.

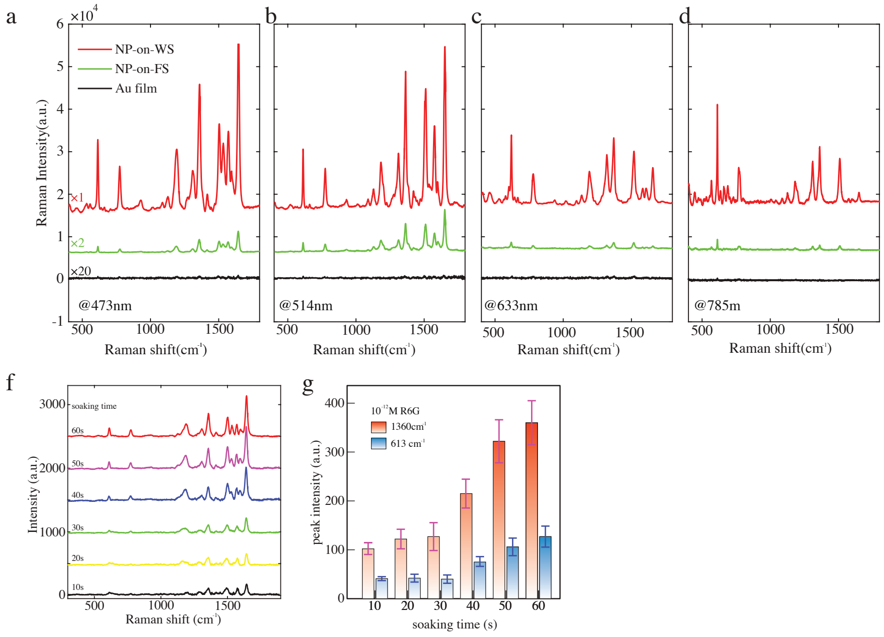

e

	473nm	514nm	633nm	785nm
EF@1360cm^{-1} (NP-on-WS)	4.41 $\times10^{9}$	4.90 $\times10^{9}$	2.47 $\times10^{9}$	1.72 $\times10^{9}$
EF@1360cm^{-1} (NP-on-FS)	0.34 $\times10^{9}$	1.54 $\times10^{9}$	0.18 $\times10^{9}$	0.36 $\times10^{9}$
α_I ($I_{NP\text{-}on\text{-}WS}/I_{NP\text{-}on\text{-}FS}$)	20.9	5.2	22.8	23.3

Supplementary Figure 23. SERS detection with sample rising process. **a-e** Broadband SERS spectra at different pump laser wavelength (a) 473nm (b) 514 nm (c) 633 nm and (d) 785nm. Both the NP-on-WS and NP-on-FS structures are measured to demonstrate the enhancement from the curvature of the substrate. The EF calculated by eq.(15) and α_I are list in (e). **f** SERS spectra with different soaking times. **g** SERS peak intensity at 613 cm^{-1} and 1360 cm^{-1} as a function of the soaking time.

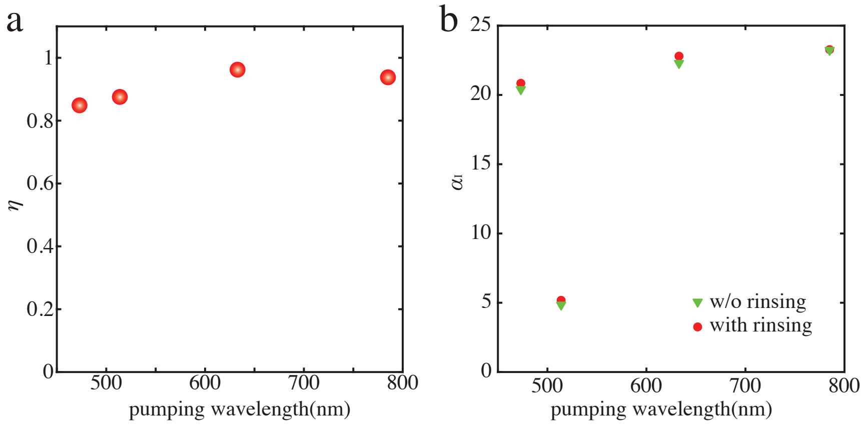

Supplementary Figure 24. **a**. The value of η at different pump laser wavelength, where η is the ratio between Raman signals at 1360 cm^{-1} with washed and unwashed sample. **b** A comparison of α_I for the samples with and without rinsing process.

Supplementary Note 15: Statistics of the nanoparticle density on flat/warped substrate

Supplementary Figure 25 summarises the statistical result of the NP density μ_M based on SEM images. The nanoparticles share the same morphology statistically, since they are formed before arriving the substrate in the cluster beam deposition process (Supplementary Note 8). Here the NP density μ_M is counted with the same cross-section area, to evaluate the number of NPs under laser spot with a unit area. Supplementary Figure 25 a and b illustrate typical NP counting for NP-on-WS and NP-on-FS separately. Here we use four different configurations wit different coverage rates of the NPs. For each configuration, we control the number of NPs on a fixed surface area to the same level, guarantee the similar averaged NPs distance. The result of the density statics is shown in Fig.S25c. In the EF calculations in the previous section, $\mu_M = 343/\mu m^2$ for NP-on-FS and $\mu_M = 557/\mu m^2$ for NP-on-WS.

Not only induces the effective permittivity gradient, the curvature also increases the NP density(1.6 times) also contributes to the enhancement of the Raman signal, as reported by many other 3D SERS systems [22, 13, 15, 1].

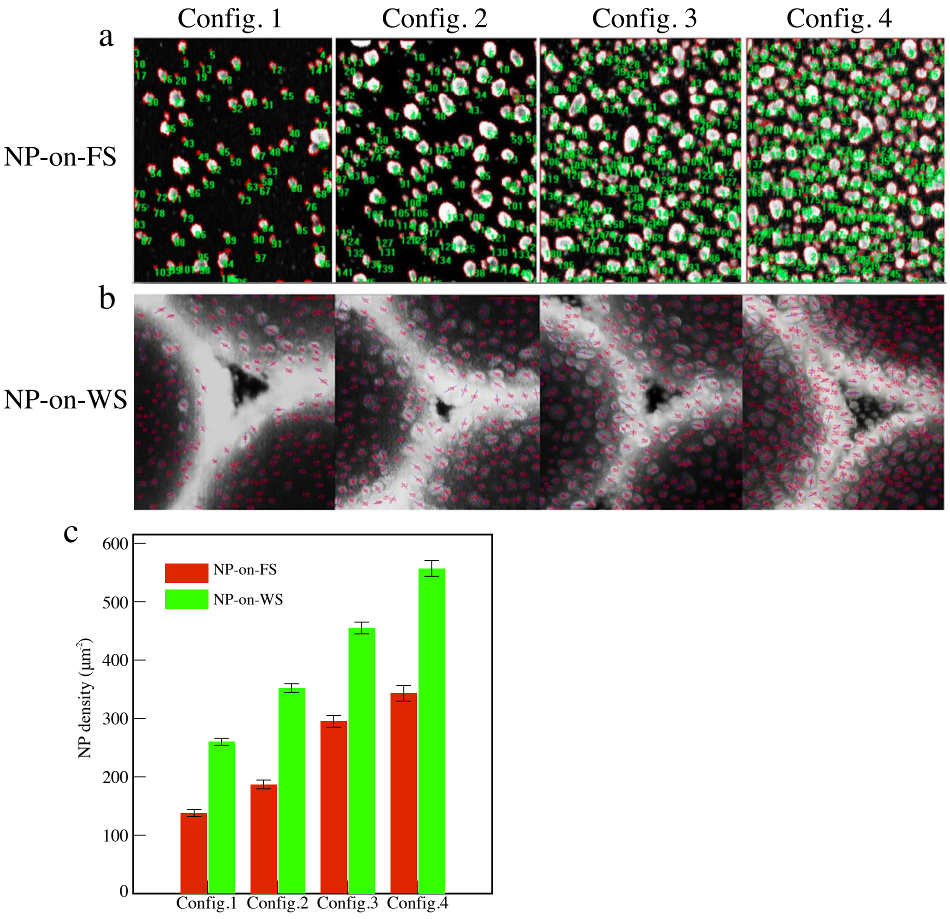

Supplementary Figure 25. **a** Typical SEM images for NP counting for NP-on-WS at different coverage rates. **b** Typical SEM images for NP counting for NP-on-FS at different coverage rates. **c** Statistics for NP density μ_M for NP-on-WS and NP-on-FS. The density is counted for fixed cross-section area instead of surface area.

Supplementary Note 16: Protein SERS indentification

By virtue of the enhancement for both field intensity and volume of the hotspots on warped substrate, our platform provides a feasible way to detect proteins, with size (tens of nanometers) beyond the volume of hotspot formed by conventional plasmonic nanostructures. Here we implement the SERS spectroscopy to two different types of proteins, the bovine serum albumin (BSA) and human epidermal growth factor receptor 2 (HER2). The former is a standard calibration protein while the later is an important biomarker and target of therapy for approximately 30% of breast cancer patients [16]. Figure 26 illustrates the SERS spectra of the two protein with different concentrations with 60s soaking time separately. The Raman peaks agree well with the previous results [2, 17, 21], unambiguously showing the fast identification of the proteins.

The proteins were purchased from Sigma-Aldrich. The molecular weight of BSA protein and HER2/c-erbB-2 peptides is 66KDa and 1428.48 Da, respectively. To investigate the effectiveness of the SERS substrates, the SERS substrate was immersed in BSA aqueous solution and HER2/c-erbB-2 aqueous solution with different concentrations (10^{-6}M, 10^{-8}M and 10^{-10}M) for 60 seconds. After drying at room temperature, it was rinsed with deionized water to remove the free protein molecules and finally dried in air at room temperature. For comparison, a drop of protein aqueous solution (1mM, $\sim$10 μL) was deposited on the surface of a silica substrate as a reference sample.

There is a challenge for the detection at the single molecule level for the proteins, mainly due to the biological issues such as protein reorganisation and denaturation. Our platform may shed light on the further improvement of the sensitivity by combing the optical design and the biological binding sites preparation for precisely capturing and aligning the proteins on the nanostructure surface [23, 6].

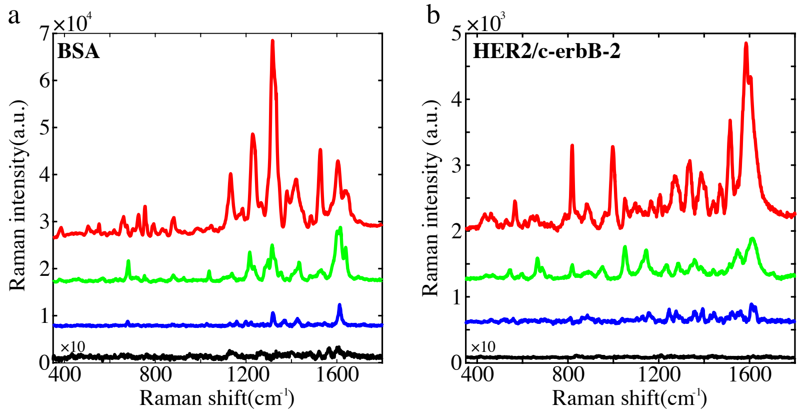

Supplementary Figure 26. Protein identification with TO inspired SERS system. SERS spectra of **a** BSA and **b** HER2 at different concentrations are demonstrated. Three different concentrations is tested with 10^{-10}M(blue), 10^{-8}M(green) and 10^{-6}M(red). The black solid line is a reference with the concentration of 1mM.

Supplementary Note 17: The evidence of enhancement induced by the substrate curvature

In the article, we implement theoretical analysis, simulations and experiments to demonstrate the field enhancement induced by curved substrate as the following:

- By first principle simulations we prove that the spatial gradient of refractive index induces field enhancement (Supplementary Note 1), and we theoretically proved that the spatial gradient in refractive index is equivalent to a curved space by transformation optics (Supplementary Note 2) .
- By using full-wave FDTD simulations, we demonstrate the field enhancement from comparisons between a curved and a flat substrate (Fig.1 in the main text, Supplementary Note 4-6).
- We implement a bunch of experiments to compare the SERS signal between a curved and a flat substrate to clarify the enhancement (Fig.3 in the main text, Supplementary Note 9,12,14-15).

Supplementary References

[1] Manohar Chirumamilla, Andrea Toma, Anisha Gopalakrishnan, Gobind Das, Remo Proietti Zaccaria, Roman Krahne, Eliana Rondanina, Marco Leoncini, Carlo Liberale, Francesco De Angelis, et al. 3d nanostar dimers with a sub-10-nm gap for single-/few-molecule surface-enhanced raman scattering. *Advanced Materials*, 26(15):2353–2358, 2014.

[2] Gobind Das, Federico Mecarini, Francesco Gentile, Francesco De Angelis, HG Mohan Kumar, Patrizio Candeloro, Carlo Liberale, Giovanni Cuda, and Enzo Di Fabrizio. Nano-patterned sers substrate: application for protein analysis vs. temperature. *Biosensors and Bioelectronics*, 24(6):1693–1699, 2009.

[3] Pablo G Etchegoin, Matthias Meyer, E Blackie, and Eric C Le Ru. Statistics of single-molecule surface enhanced raman scattering signals: Fluctuation analysis with multiple analyte techniques. *Analytical chemistry*, 79(21):8411–8415, 2007.

[4] H Galinski, G Favraud, H. Dong, J S Totero Gongora, G Favaro, M Döbeli, R Spolenak, A Fratalocchi, and F Capasso. Scalable, ultra-resistant structural colors based on network metamaterials. *Light Sci. Appl.*, 6:e16233, 2017, doi: 10.1038/lsa.2016.233.

[5] Manas R Gartia, Zhida Xu, Elaine Behymer, Hoang Nguyen, Jerald A Britten, Cindy Larson, Robin Miles, Mihail Bora, Allan SP Chang, Tiziana C Bond, et al. Rigorous surface enhanced raman spectral characterization of large-area high-uniformity silver-coated tapered silica nanopillar arrays. *Nanotechnology*, 21(39):395701, 2010.

[6] Xiao-Peng He, Xiu-Wen Wang, Xiao-Ping Jin, Hao Zhou, Xiao-Xin Shi, Guo-Rong Chen, and Yi-Tao Long. Epimeric monosaccharide- quinone hybrids on gold electrodes toward the electrochemical probing of specific carbohydrate- protein recognitions. *Journal of the American Chemical Society*, 133(10):3649–3657, 2011.

[7] F. Hecht. New development in freefem++. *J. Numer. Math.*, 20(3-4):251–265, 2012.

[8] Jian-An Huang, Ying-Qi Zhao, Xue-Jin Zhang, Li-Fang He, Tai-Lun Wong, Ying-San Chui, Wen-Jun Zhang, and Shuit-Tong Lee. Ordered ag/si nanowires array: wide-range surface-enhanced raman spectroscopy for reproducible biomolecule detection. *Nano letters*, 13(11):5039–5045, 2013.

[9] Andrzej Kudelski. Raman studies of rhodamine 6g and crystal violet sub-monolayers on electrochemically roughened silver substrates: Do dye molecules adsorb preferentially on highly sers-active sites? *Chemical Physics Letters*, 414(4-6):271–275, 2005.

[10] A. J. Labelle, M. Bonifazi, Y. Tian, C. Wong, S. Hoogland, G. Favraud, G. Walters, B. Sutherland, M. Liu, Jun Li, Xixiang Zhang, S. O. Kelley, E. H. Sargent,

and A. Fratalocchi. Broadband epsilon-near-zero reflectors enhance the quantum efficiency of thin solar cells at visible and infrared wavelengths. *ACS Appl. Mater. Interfaces*, 9:5556–5565, 2017. PMID: 28156089.

[11] N. Landy, Y. Urzhumov, and D. R. Smith. Quasi-conformal approaches for two and three-dimensional transformation optical media. In H. D. Werner and D.-H. Kwon, editors, *Transformation Electromagnetics and Metamaterials.* Springer-Verlag, London, 2014.

[12] EC Le Ru, E Blackie, Matthias Meyer, and Pablo G Etchegoin. Surface enhanced raman scattering enhancement factors: a comprehensive study. *The Journal of Physical Chemistry C*, 111(37):13794–13803, 2007.

[13] Seunghyun Lee, Myung Gwan Hahm, Robert Vajtai, Daniel P Hashim, Theerapol Thurakitseree, Alin Cristian Chipara, Pulickel M Ajayan, and Jason H Hafner. Utilizing 3d sers active volumes in aligned carbon nanotube scaffold substrates. *Advanced Materials*, 24(38):5261–5266, 2012.

[14] Ulf Leonhardt. Optical conformal mapping. *Science*, 312:1777–1780, 2006.

[15] Honglin Liu, Zhilin Yang, Lingyan Meng, Yudie Sun, Jie Wang, Liangbao Yang, Jinhuai Liu, and Zhongqun Tian. Three-dimensional and time-ordered surface-enhanced raman scattering hotspot matrix. *Journal of the American Chemical Society*, 136(14):5332–5341, 2014.

[16] Zahi Mitri, Tina Constantine, and Ruth O'Regan. The her2 receptor in breast cancer: pathophysiology, clinical use, and new advances in therapy. *Chemotherapy research and practice*, 2012, 2012.

[17] Hyejin Park, Sangyeop Lee, Lingxin Chen, Eun Kyu Lee, Soon Young Shin, Young Han Lee, Sang Wook Son, Chil Hwan Oh, Joon Myong Song, Seong Ho Kang, et al. Sers imaging of her2-overexpressed mcf7 cells using antibody-conjugated gold nanorods. *Physical Chemistry Chemical Physics*, 11(34):7444–7449, 2009.

[18] J. B. Pendry, D. Schurig, and D. R. Smith. Controlling electromagnetic fields. *Science*, 312:1780–1782, 2006.

[19] D. Schurig. An aberration-free lens with zero f-number. *New J. Phys.*, 10:115034, 2008.

[20] Yi Tian, Francisco Pelayo García de Arquer, Cao-Thang Dinh, Gael Favraud, Marcella Bonifazi, Jun Li, Min Liu, Xixiang Zhang, Xueli Zheng, Md. Golam Kibria, Sjoerd Hoogland, David Sinton, Edward H. Sargent, and Andrea Fratalocchi. Enhanced solar-to-hydrogen generation with broadband epsilon-near-zero nanostructured photocatalysts. *Adv. Mater.*, 29:1701165–n/a, 2017. 1701165.

[21] Li Zhang, Changrong Guan, Ying Wang, and Jianhui Liao. Highly effective and uniform sers substrates fabricated by etching multi-layered gold nanoparticle arrays. *Nanoscale*, 8(11):5928–5937, 2016.

[22] Qi Zhang, Yih Hong Lee, In Yee Phang, Choon Keong Lee, and Xing Yi Ling. Hierarchical 3d sers substrates fabricated by integrating photolithographic microstructures and self-assembly of silver nanoparticles. *Small*, 10(13):2703–2711, 2014.

[23] Xianfeng Zhang, Xuezhong Du, Xuan Huang, and Zhongpeng Lv. Creating protein-imprinted self-assembled monolayers with multiple binding sites and biocompatible imprinted cavities. *Journal of the American Chemical Society*, 135(25):9248–9251, 2013.
